# Drug screening in zebrafish larvae reveals inflammation-related modulators of secondary damage after spinal cord injury in mice

**DOI:** 10.7150/thno.81332

**Published:** 2023-04-23

**Authors:** Ana-Maria Oprişoreanu, Fari Ryan, Claire Richmond, Yuliya Dzekhtsiarova, Neil O. Carragher, Thomas Becker, Samuel David, Catherina G. Becker

**Affiliations:** 1 Centre for Discovery Brain Sciences, University of Edinburgh, The Chancellor's Building, 49 Little France Crescent, Edinburgh, EH16 4SB, UK; 2 Center for Regenerative Therapies Dresden, TU Dresden, Fetscherstraße 105, 01307 Dresden, Germany; 3 Centre for Research in Neuroscience, Research Institute of the McGill University Health Centre, 1650 Cedar Ave., Montreal, Quebec, H3G 1A4; 4 Cancer Research UK Edinburgh Centre, Institute of Genetics and Cancer, University of Edinburgh, Edinburgh, EH4 2XR, UK

**Keywords:** chronic inflammation, histamine receptors, zebrafish, irf8, Betazole, Bortezomib, Sildenafil, Cimetidine

## Abstract

Prolonged inflammation after spinal cord injury is detrimental to recovery. To find pharmacological modulators of the inflammation response, we designed a rapid drug screening paradigm in larval zebrafish followed by testing of hit compounds in a mouse spinal cord injury model.

**Methods:** We used reduced *il-1β* linked green fluorescent protein (GFP) reporter gene expression as a read-out for reduced inflammation in a screen of 1081 compounds in larval zebrafish. Hit drugs were tested in a moderate contusion model in mice for cytokine regulation, and improved tissue preservation and locomotor recovery.

**Results:** Three compounds robustly reduced *il-1β* expression in zebrafish. Cimetidine, an over-the-counter H2 receptor antagonist, also reduced the number of pro-inflammatory neutrophils and rescued recovery after injury in a zebrafish mutant with prolonged inflammation. Cimetidine action on *il-1β* expression levels was abolished by somatic mutation of H2 receptor *hrh2b*, suggesting specific action. In mice, systemic treatment with Cimetidine led to significantly improved recovery of locomotor behavior as compared to controls, accompanied by decreased neuronal tissue loss and a shift towards a pro-regenerative profile of cytokine gene expression.

**Conclusion:** Our screen revealed H2 receptor signaling as a promising target for future therapeutic interventions in spinal cord injury. This work highlights the usefulness of the zebrafish model for rapid screening of drug libraries to identify therapeutics to treat mammalian spinal cord injury.

## Introduction

Spinal cord injury (SCI) in humans can lead to permanent loss of sensory and motor functions, due to wide-spread tissue damage and insufficient repair. Primary tissue damage is caused directly by the impact, and secondary damage that occurs over a wider area ensues as a consequence of the break-down of the blood-spinal cord barrier 1-3. This is often accompanied by chronic inflammation 4, 5. While the immune response can have beneficial effect on tissue repair it can also exacerbate tissue damage, depending on the phenotype or state of activation of immune cells. After SCI, immune cells largely display a pro-inflammatory state, including release of Il-1β, which negatively affects recovery after spinal injury in mammals and zebrafish 6-8. This polarization of mainly macrophages and microglial cells is often termed M1 (pro-inflammatory) or M2 (pro-regenerative) with many mixed activation states observed *in vivo* 9.

The only drug in regular clinical use that targets the immune system after SCI is Methylprednisolone 10, which generally suppresses the immune response. However, as a glucocorticoid it has a number of side effects and is therefore of limited benefit to patients for functional recovery 11. Therefore, it is desirable to find drugs that can modulate the immune response in a way that enhances tissue sparing and allows functional repair. To this end, we turned to an unbiased (drug-target agnostic) but mechanistically informed (Il-1β reporter) image-based phenotypic screening approach in spinal-injured larval zebrafish.

Larval zebrafish are the foremost vertebrate whole-organism screening tool 12, 13 and larval and adult zebrafish can be cost-effectively screened for effects of drug and genetic manipulations on the outcome of spinal cord injury 14-16. That is owed to the small size of larvae and the possibility to automate the process by using microfluidic handling machines and automated imaging 17, 18. Moreover, larval zebrafish are transparent and allow for fluorescent transgenes to be used as precise and rapid screening assay read-outs.

Larval and adult zebrafish, in contrast to mammals, are capable of functional spinal cord regeneration 19, 20. However, regeneration depends on a pro-regenerative immune response and can be accelerated by manipulations of the immune reaction 8. The innate immune response in zebrafish shares hallmark features with that in mammals 21, 22. After spinal injury in larvae, neutrophils are the first responders to a spinal injury, followed by macrophage and microglia, which are necessary to induce a sharp decline in neutrophil numbers 8. During the early phase of the immune response there is a sharp peak in the expression of *il-1β* and *tnf*, followed by anti-inflammatory *tgfs* 8, 23. This response is controlled by macrophages. In a macrophage-less mutant for the macrophage differentiation gene *irf8* 24, pro-inflammatory cytokines and neutrophils prevail at the injury site for a long time and repair is permanently impaired 8. Prolonged inflammation after injury is seen in the *irf8* mutant and in mammals 25, 26. However, there are also clear differences between larval zebrafish and mammals. For example, the adaptive immune system only develops at post-larval stages in zebrafish, such that zebrafish larvae allow studying the innate immune response in isolation 27.

Here we used a fluorescent reporter zebrafish line for *il-1β* to screen a panel of drugs for potentially beneficial immune modulators of SCI 28. We found that the histamine receptor 2b antagonist Cimetidine suppressed *il-1β* expression in larval zebrafish and partially rescued impaired regeneration in the *irf8* zebrafish mutant model for chronic inflammation. Importantly, in a mouse model of moderate spinal cord contusion injury, the drug modulated the immune response and led to significant tissue sparing and improved functional recovery. This study highlights the usefulness of the zebrafish SCI model to rapidly screen drug libraries for potential therapeutics to treat mammalian SCI for which there is currently no effective treatment.

## Materials and Methods

### Zebrafish experiments

#### Animals

Zebrafish used in this work were: WIK wild-type strain, Tg(*il-1β*:eGFP)^sh445^, abbreviated as *il-1β*:GFP 28, *irf8*^st95/^ abbreviated as *irf8-/-* 24, Tg(*Xia.Tubb*:DsRed)^zf1426^, abbreviated as *Xia.Tubb*:DsRed^29^ and Tg(*mpeg1.1:*EGFP)^gl22^, abbreviated as *mpeg1*:GFP 30. All fish strains were kept and bred according to standard procedures. Zebrafish experiments were performed under British Home Office licence PP8160052 and TVV 36/2021 State of Saxony.

#### Spinal cord injury

At 3 days post-fertilization (dpf) zebrafish, larvae were anesthetized in PBS containing 0.02% MS-222. Larvae were transferred to an 4% agarose-coated Petri dish, placed in a lateral position and the excess water removed. The manual spinal cord injury was done using a 30G syringe needle at the level of the 15^th^ myotome while leaving the notochord intact, as previously described 31. Briefly, under transmitted light the dorsal edge of the notochord was used as a landmark to indicate the lower edge of the injury. Larvae that were inadvertently under- or over-lesioned (notochord damaged), were excluded from the experiment at this point. Thereafter, larvae were transferred to a dish or 24-well plate containing fish water or appropriate chemical compound.

#### Compound screening

At 3 dpf larvae were pre-incubated for 4h with 10µM of chemical compounds in fish water (E3 medium) before a spinal cord injury was performed as described below. Pre-incubation was used to exclude any diffusion delays. Following the spinal cord injury, the larvae were further incubated in the same chemical compounds for 16-18h. The drug treatment was performed in 24-well plates, with 6 larvae per well. For the prescreening step, 6 larvae per compound were used, while for the validation step between 10 and 12 larvae were used. Chemical compounds used in this screening were from Enzo Screen-Well^TM^ FDA Approved Drug Library V2 (Cat. no. BML 2843-0100, 786 compounds) and ApexBio anti-inflammatory library (DiscoveryProbe^TM^ Immunology/Inflammation Compound Library, Cat. No. L1042, 295 compounds). The stock concentration of the compounds was at 10 mM in DMSO and thus the final DMSO concentration in fish water was 0.1% DMSO. Vehicle controls were also at 0.1% DMSO. For dose-dependence experiments and bridging assays, compounds were purchased individually and solubilized according to the manufacturer's instructions (usually 10 or 50 mM in DMSO): cimetidine (ApexBio, cat. no. APE-B1557), sildenafil citrate (ApexBio, cat no. APE-A4321), Betazole (ChemScene, cat. no. CS-0013438).

#### Image acquisition and GFP fluorescence quantification for screening

Larvae were fixed in 4% paraformaldehyde for 3-4 h, washed in PBS-0.1% Triton X-100 and stored in 70% glycerol overnight. For imaging, the decapitated larvae were mounted on a glass coverslip with 70% glycerol. Images were acquired using a Zeiss AxioImager Z1 with ApoTome.2 upright microscope and an inverse Zeiss Imager microscope with an ApoTome unit (LD LC1 Plan-Achromat 20x-objective NA = 0.8).

To quantify the overall GFP fluorescence signal, the open-source Fiji software was used. First the z-stacks of each imaged larvae (Z-step = 1 µm) were converted into maximum intensity projections, followed by background subtraction (rolling ball radius = 1/3 of the image width) and mean intensity calculation. The quantification of the overall GFP signal was performed using the entire depth of the injury site (imaging depth = 80-90 µm). Images were blinded for analysis.

#### Assessment of the axonal bridging

To assess the presence of the axonal bridge that connects the rostral to the caudal spinal cord end the injured larvae were analyzed at 48 hours post-lesion (hpl) under a fluorescent stereo-microscope (Leica M165 FC), according to established procedures 8, 32. For the analysis the observer was blinded to the genotype and treatment of the larvae.

#### Assessment of behavioral recovery

To analyze the swimming behavior the ZebraBox from ViewPoint Behavior Technology (Lyon, France) was used. Larvae (5 dpf) were arrayed in 24-well plates, with one larva per well (different conditions were arrayed randomly in the plate). The plates were kept in the incubator for at least 1 h before testing for the larvae to acclimatize. Larval movements were automatically tracked for 30 s. For the first 10 s of tracking a series of vibration stimuli were applied (10 x ON, 10 x OFF; 500 ms each; frequency was set at 600 [target power 100]). IWR-1 served as positive control for impaired recovery 32. The total swimming distance was computed automatically, with the graph and statistics generated in GraphPad.

#### Zebrafish RNA extraction and qRT-PCR

RNA was extracted from 3 dpf larvae at 4 hpl from cimetidine and vehicle-treated groups (50 larvae/group/experiment) using the RNeasy Mini Kit (Qiagen) according to the manufacturer's instructions. We enriched the injury sites by cutting away rostral and caudal parts from the larvae. Genomic DNA was removed by using Turbo DNA-free kit (AM1907, Invitrogen), according to manufacturer's instructions and PCR primers were designed using the Primer-BLAST website from NCBI with preference on an exon-exon junction set to on. cDNA was synthesized using the iScript™ cDNA Synthesis Kit (BioRad, cat. no. 1708890) according to the manufacturer's instructions. Reverse transcription-PCR was performed with the following set of primers: il-1β - fw 5' ATGGCGAACGTCATCCAAGA 3' and rv 5' GAGACCCGCTGATCTCCTTG 3', analyzed-actin fw 5' CACTGAGGCTCCCCTGAATCCC 3' and rv 5' CGTACAGAGAGAGCACAGCCTGG 3', hrh2a - fw 5' CACGCCCATTCTTCAAAAGG 3' and rv 5' CCATTTGTAGCGCTGTTATCGTC 3', and hrh2b - fw 5' GCTGCTGAAAGCGTTTGGAA 3' and rv 5' GAATACGCCGCACCTGTTC 3' 33. qRT-PCR was performed using the SsoAdvanced Universal SYBR Green Supermix kit (BioRad) according to the manufacturer's instructions and run in triplicates on a LightCycler 96 instrument (Roche). The data was analyzed using the LightCycler 96 application software v1.1 (Roche).

#### Hybridization chain reaction (HCR) and quantification

The hybridization chain reaction (HCR) for *il-1β* was performed using a previously published protocol 34. Briefly, the larvae (4 hpl) were fixed for 4h in 4% PFA, followed by over-night storage in methanol at -20 °C. The next day the samples were rehydrated and permeabilized using proteinase K (10µg/ml in 1xPBST) for 45 min. For the hybridization, 3 pmol of *il-1β* probe set was used at 37 °C overnight. The following day the HCR amplification was performed using the B3-647 amplifier (Molecular Instruments). Following the HCR amplification, the samples were extensively washed in 5x SSCT and 1x PBST buffer and stored in 70% glycerol.

For the quantification of the RNA puncta the open-source Fiji software was used. First, all the images were converted to maximum intensity projections (MIPs), followed by background subtraction (ball radius 1/3 of image height). Next, maximum intensity was automatically calculated, and then the threshold for each image was set (20% of the maximum intensity), generating a binary image. From this image the number of puncta was calculated using the Analyse Particle function.

#### Immunohistochemistry and neutrophil counting

Following the spinal cord injuries, the larvae were fixed at 2 and 24 hpl with 4% PFA for 3-4h at room temperature. The larvae were rinsed 3x5 min with PBS - 0.1%Tween (PBST), followed by methanol fixation overnight. The next day, the larvae were rehydrated using decreasing concentration of methanol, followed by PBST rinsing and proteinase K treatment (10 µg/ml) in PBST for 45 min at room temperature. After this step, the larvae were refixed in 4%PFA for 15 min at room temperature, washed with PBST and blocked using blocking buffer (1% BSA, 1% DMSO, 1% normal donkey serum, 0.5% Triton X-100 in PBS, pH. 7.4) for 1-2h at room temperature. The larvae were then incubated for 2 nights at 4°C with the primary antibodies in blocking buffer. The primary antibodies were chicken anti-GFP (1:200, ab13970) and rabbit anti-mpx (1:100, GeneTex GTX128379). The primary antibodies were washed off, and samples incubated with secondary antibodies overnight. The secondary antibodies were AlexaFlour488 donkey anti-chicken (Jackson ImmunoResearch, 1:200) and AlexaFlour647 donkey anti-rabbit, (Jackson ImmunoResearch, 1:200). After this the samples were washed with PBST and kept in 70% glycerol at 4°C until imaging. The image acquisition of the samples was done using an inverted Zeiss LSM 980 confocal microscope (CMCB Light Microscopy Facility of the CMCB Technology Platform, TU Dresden). The number of neutrophils in the vicinity of the injury site was counted in a volume of interested that was centered on the lesion site (width, X-axis - 200 µm, height, Y-axis - 75 µm, depth, Z-axis - 50 µm). The number of cells was counted manually in Zen software using the 3D view as well. For the analysis the observer was blinded to the conditions of the experiment using the Blind analysis Tool v1.0 plugin in Fiji software (randomization and encryption of the files).

#### Macrophage counting and intensity measurements

Macrophages at the injury site were counted in the same way as neutrophils (above). For intensity measurement the analysis was performed in Fiji. The images were split into individual channels, and background subtraction was performed. Next, using the red (macrophage) channels an outline mask was generated, which was applied over the GFP (*il-1β*) channel to calculate the mean grey value.

#### Crispr/Cas9 design, injection and RFLP analysis

The CrRNAs targeting the *hrh2a* and *hrh2b* genes were designed such that the restriction enzyme recognition site was overlapping the Cas9 cutting site, as previously described (Keatinge et. al., 2021). RNA oligos were resuspended at 2 nmol/100 µL water and stored at -20C. For injections 1 µl sCrRNA and 1 µl TracrRNA (Sigma, cat. no. TRACRRNA05N) were incubated for 5 min at 95°C, followed by cooling on ice for 20 min. 1 µl Cas9 (NEB, cat. no. M0386M) and 1 µl Phenol Red was added to the mix and further incubated for 5-10 min at 27°C.

The CrRNA target site for* hrh2a* and *hrh2b* genes were: 5' TGCTTCATCGTTTCGCTAG 3' and 5' CCGCTCTTCTATCCCAGGA 3'.

To analyze the efficiency of the CrRNAs by restriction fragment length polymorphism (RFLP), genomic DNA was extracted from 24 - 48 hours post-fertilization (hpf) embryos by boiling the single zebrafish embryos in 100 µl 50mM NaOH for 5 min, followed by the addition of 10 µl Tris-HCl pH 8.0. The PCR was performed using the MyTaq HS Red Mix according to the manufacturer's instructions with the following pairs of primers: *hrh2a* fw 5' CTGGTCTGTCTGGCTGTGTA 3' and rev 5' GCATAGCATGACGTCCAGTG 3', and *hrh2b* fw 5' CTGGCCATTAGTGTTGACCG 3' and rev 5' CTTCCTCTCCCTCATCCCAC 3'. For each CrRNA, RFLP was done by adding to the final PCR product 1 µl of the respective restriction enzyme (NheI for *hrh2a* and BstNI for *hrh2b*) and incubated for 1-2 h at the optimal temperatures for each restriction enzyme. The RFLP analysis was performed on 4 uninjected samples and 8 injected samples.

### Mouse experiments

#### Spinal cord injury and Cimetidine treatment

Female C57BL/6 mice (8 weeks of age) were deeply anesthetized using ketamine/xylazine/acepromazine (50:5:1 mg/kg) and a partial laminectomy performed at vertebral level T11. Spinal cord contusion injury was done on the exposed region using the Infinite Horizons spinal cord impactor (Precision Scientific). All contusions were moderate injuries with impact force of 50 kdyn and tissue displacement of 400-600 μm. Alternate animals were assigned to experimental and control groups immediately after surgery (*n* = 11 mice per group). The experimental group was treated twice a day intraperitoneally (IP) with Cimetidine 15 mg/kg body weight starting immediately after injury until day 14. Mice in the control group were injected with vehicle (saline) IP. Mice were euthanized at 28 days post-injury and the tissue processed for histological analysis as described below. Sample size was calculated based on our historical experience with BMS analysis for medium effect size (f=0.25), a confidence coefficient of 0.05 and a statistical power of 0.80. All protocols were approved by the McGill University Animal Care Committee and followed the guidelines of the Canadian Council on Animal Care.

#### Locomotor analysis

Locomotor outcome after spinal cord contusion injury was assessed using the Basso Mouse Scale (BMS) 35. Mice (*n* = 11 per experimental and control groups) were scored in an open-field environment by two individuals blinded to experimental conditions and trained in BMS analysis in Dr. Michelle Basso's laboratory at Ohio State University. Consensus scores for each animal were averaged at each time point for a maximum of 9 points for the BMS score. Locomotor performance was also assessed at day 28 using the DigiGait system (Mouse Specifics, Quincy, MA) at a speed of 10cm/s. Only animals used for locomotor analysis were used for histological analysis at day 28.

#### Histological analysis

Mice (*n* = 5-6 per group) were deeply anesthetized and perfused intracardially with 4% paraformaldehyde in 0.1 M phosphate buffer (PB). A 5 mm length of spinal cord was removed, centered on the injury, postfixed for 24 h, cryoprotected in 30% sucrose in 0.1 M phosphate-buffered saline (PBS) for 48 h and serial 14 μm cryostat sections picked-up on glass slides. Tissue sections were incubated with blocking solution containing 0.2% Triton X-100 (Sigma Millipore), 2% ovalbumin (Sigma Millipore), and 5% normal goat serum (Jackson ImmunoResearch Laboratories) in 0.01M PBS for 3 h at room temperature to block nonspecific binding. The following primary antibodies were used for immunofluorescence labeling: guinea pig polyclonal anti-NeuN (for neurons; 1:300; Synaptic Systems, 266 004); rabbit polyclonal anti-GFAP (for astrocytes; 1:1500; Dako, Z0334); rat monoclonal anti-5-HT (1:100; Abcam, ab6336); rabbit anti-histamine H2 Receptor (HRH2, 1:100; Alomone labs, AHR-002;); rabbit anti-Histidine Decarboxylase (HDC, 1:1000, Progen, 16045); rabbit anti-IL-1β (1:100; abcam, ab283818); mouse anti-neurofilament 200 (1:300, Sigma-Aldrich, N0142), rabbit anti-4-HNE (1:100; Abcam; ab46545); rat anti-GFAP (for astrocytes; 1:500; Invitrogen, 13-0300). Tissue sections were incubated with appropriate fluorescent conjugated secondary antibodies: anti-guinea pig Alexa Fluor 568, anti-rabbit Alexa Fluor 488, anti-rat Alexa Fluor 568 (all 1:500, Invitrogen) or anti-guinea pig Alexa Fluor 647. Tissue sections were stained with Luxol Fast Blue (LFB) to assess myelin sparing. For myelin staining, sections were first dehydrated in 50%-95% ethanol and then incubated in a 0.1% LFB solution overnight at 37°C. Next day, the slides, after cooling at 4°C for 1 h, were incubated in 0.05% lithium carbonate solution, followed by differentiation in ethanol and then processed in xylene and cover-slipped with Entellan (Electron Microscopy Sciences). Images of the whole cross-section of spinal cords were taken using an Axioskop2 plus microscope (Carl Zeiss) using Bioquant image analysis software (Bioquant life Sciences).

#### Quantification of mouse histology data

Serial mouse spinal cord sections were cut through 3 mm length. The center of injury in cryostat sections in all spinal cords was identified using slides stained with Nissl stain. The number of NeuN+ neurons in the ventral horn was quantified from single plane confocal images taken at regular distances either side of the center. Lesion area was estimated in sections stained with anti-GFAP and estimated as a percentage of the cross-section of the spinal cord. Myelin sparing was measured using LFB-stained cross-sections of the spinal cord using the ImageJ/Fiji software (version 2.3.0/1.53q, National Institutes of Health). Spared myelin was measured using the ImageJ Threshold feature, this value was divided by the whole cross-sectional area of spinal cord to obtain the percentage of spared myelin. Data were analyzed by two-way repeated-measures (RM)-ANOVA with post hoc Bonferroni tests.

To estimate serotonergic innervation, fluorescence intensity (as measured by Integrated Density [IntDen]) of 5-HT labeling of the ventral horn, 1 mm caudal to the injury site, measured separately in the right and left ventral horns, was quantified using the ImageJ software. Fluorescence intensity of HHR2 and HDC was quantified in right and left dorsal horns of uninjured animals and at 500 μm caudal to the epicenter 48 h after SCI in vehicle-treated and cimetidine-treated animals. Fluorescence intensity of IL-1β was quantified in right and left half section of spinal cord of uninjured animals and at the epicenter 48h after SCI in vehicle-treated and cimetidine-treated animals. The data of right and left side was averaged for each animal. Fluorescence intensity of 4-HNE was quantified from confocal images taken at the center 4 weeks after injury, in SCI-vehicle and SCI-cimetidine animals and in uninjured mice. Changes in the expression of HHR2, HDC, IL-1β and 4-HNE markers were analyzed by using one-way ANOVA with Tukey post-hoc comparisons*.* The number of CD11b+ cells with DAPI stained nuclei in the lesion site were manually counted from confocal images taken at the epicenter. The staining intensity of CD11b was also quantified as IntDen. The results were analyzed by Mann-Whitney-U test for single comparison between two groups. All data are plotted as mean ± SEM unless otherwise specified.

#### RNA isolation and QRT-PCR

Total RNA was extracted from the mouse spinal cord 1day after SCI using RNeasy Mini Kit (Qiagen) following manufacturer's instructions. cDNA was reverse transcribed with the Qiagen Quantitect Kit (Cat. No. 205311). Reverse transcription-PCR was performed using SYBR Green PCR Master Mix (Applied Biosystem) and ABI OneStep cycler (Applied Biosystems) using the following set of primers for *il-1β*: fw 5' ATGGGCAACCACTTACCTATTT and rv 5' GTTCTAGAGAGTGCTGCCTAATG. Peptidylprolyl isomerase A (ppia) was used as an internal control using the following set of primers: Fw 5' ATGTGCCAGGGTGGTGACTTTA and rv 5' TGTGTTTGGTCCAGCATTTGCC. The results were quantified using the ΔΔCT method following standardization relative to *ppia* 36.

#### Screening for chemokine and cytokine genes

Mice were treated with 15mg/kg Cimetidine or vehicle (control) for 10 days intraperitoneally. A 3 mm length of the spinal cord centered on the lesion site was collected for analysis. Total RNA was extracted using RNeasy plus universal kit (Cat. No. 73404) followed by reverse transcription with the RT2 first strand kit (Cat. No. 330401) followed by RT2 profiler cytokine array (PAMM-150Z) according to the manufacturer's instructions (Qiagen). We screened 84 inflammation associated genes, and the data obtained were analyzed with the online Qiagen analysis software - RT^2^ profiler PCR array data analysis V3.5.

### Statistical analysis

All data were analyzed for normality and statistical tests were chosen appropriately as parametric or non-parametric. For multiple comparisons, ANOVA with post-hoc Tukey multiple comparison test or the Kruskal-Wallis test with Dunn's multiple tests was used. For single comparisons, unpaired t-test, or Mann-Whitney tests were used as appropriate (zebrafish data). Statistical comparison between groups (mouse data) were assessed by two-way repeated measures (RM)-ANOVA, followed by Bonferroni *post hoc* test. All statistical analysis and graphs were done using GraphPad Prism 8/9 software. The error bars in the graphs indicate the standard error of the mean (± SEM). A value of p ≤ 0.05 was considered significant. All analyses were conducted blinded to the experimental or control groups.

## Results

### Screening in larval zebrafish spinal injury reveals Cimetidine as a modulator of lesion-induced inflammation

**Figure 1:**
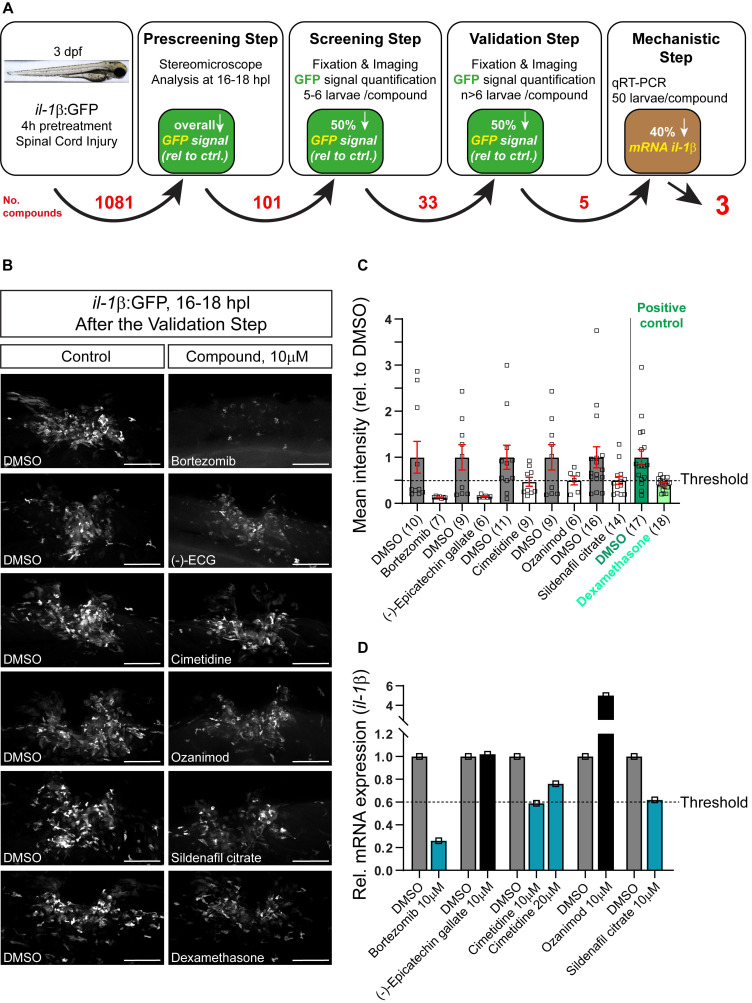
** Screening of an Il-1β reporter fish reveals drugs that reduce endogenous Il-1β mRNA expression. A:** Schematic representation of the drug screening workflow. Briefly, 3 dpf larvae were pre-treated with various compounds for 4 hours, followed by manual injury. Next day, larvae that showed an overall decrease in GFP signal (pre-screening) were fixed, fluorescence was quantified (screening step) and positive compounds were re-tested (validation) and mechanistically tested by qRT-PCR for *il-1β* mRNA. The numbers of tested compounds after each screening step are depicted in red. **B:** Representative images of 16-18 hpl drug-treated larvae are shown, including negative (DMSO) and positive control (dexamethasone). **C:** Quantification of the overall GFP fluorescence signal relative to DMSO-treated control is shown for the validation step. All five compounds elicit a ≥50% drop in the overall GFP signal. Dexamethasone treatment (positive control) induced an approx. 50% decrease in overall GFP signal versus control. **D:** Three out of five compounds lead to a reduction in the relative *il-1β* mRNA expression in wild-type larvae (50 treated or control larvae were pooled per measurement). Cimetidine (10 µM) and sildenafil citrate lead to a 40% reduction in *il-1β* mRNA expression, while bortezomib leads to a 70% reduction. Scale bars: 100 µm

Three day post-fertilization, *il-1β*:GFP larvae (transgenic fluorescent reporter fish for *il-1β* expression) were pre-incubated for 4h in various chemical compounds, followed by manual spinal cord injury and post-injury treatment with the same compound. Two small molecule compound libraries, one comprising FDA-approved compounds and one tailored to inflammation (1081 drugs in total; see Material and Methods) were tested (See Figure 1A for a schematic overview of the screening process). Next day, all treated embryos were visually inspected for GFP fluorescence in the injury site using a stereo-microscope. Only larvae that showed a visible and thus robust decrease in the overall fluorescence for a certain compound (101 compounds; Table S1) in this rapid assay were fixed, imaged and average GFP fluorescence intensity at the injury site was measured in ImageJ. Drugs that showed at least 50% reduction in the injury-induced GFP fluorescence (33 compounds; the threshold was previously established using dexamethasone as a positive control) were re-tested. If the results were reproducible after the 2^nd^ round of screening, which was the case for 5 compounds (Figure 1B,C), qRT-PCR was performed to confirm compound effects on endogenous *il-1β* mRNA levels. Eventually, three compounds were found to reduce endogenous *il-1β* mRNA expression by at least 40%. In order of effectiveness, these were Bortezomib, Cimetidine, and Sildenafil citrate (Figure 1D). Independent re-testing of the top three compounds confirmed effects of Bortezomib and Cimetidine, but not Sindenafil on *il-1β* mRNA levels (Figure S1A-C).

Bortezomib is a proteasome inhibitor with antineoplastic activity tested for treatment in multiple myeloma and other hematological and solid tumors. However, its clinical usefulness is limited because it induces painful peripheral neuropathy 37. Studies in mice showed that Bortezomib induces morphological and neurophysiological evidence of nerve damage, including axonal degeneration and painful peripheral neuropathy 38, 39. For these reasons and because effects of Sindenafil were less robust, we focused our further validation on the second-most effective drug, the H2 receptor antagonist Cimetidine. We found that concentrations of Cimetidine between 10 and 50 µM were similarly effective in reducing mRNA (Figure 1D) or reporter gene expression (Figure 4A) for *il-1β*. Moreover, Cimetidine also reduced *in situ* detectability of *il-1β* mRNA by HCR after injury (Figure S1D,E). In unlesioned larvae, *il-1β*:GFP was undetectable with and without treatment with Cimetidine (Figure S1F,G).

### Cimetidine reduces the neutrophil response to injury

**Figure 2:**
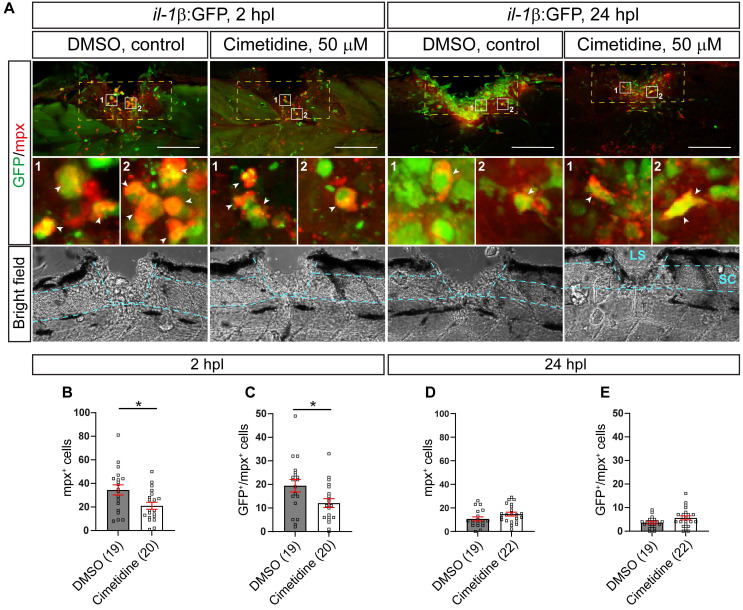
** Cimetidine reduces the number of neutrophils in the injury site. A:** Representative images of the lateral view of the injury site in the *il-1β*:GFP line treated with cimetidine and DMSO-control are shown. Broken blue lines outline spinal cord (SC) and lesion site (LS). **B, C:** At 2 hpl, the total number of neutrophils (B, mpx+, t-test, p* = 0.0143), and the number of double positive neutrophils (C, GFP^+^/Mpx^+^ t-test, p* = 0.0279) are reduced by cimetidine treatment. **D, E:** At 24 hpl, the number of double-positive cells is not altered by cimetidine treatment. Broken yellow lines outline the area of quantification. Scale bar: 100 µm.

One of the major features of a pro-inflammatory reaction to an injury is invasion of *il-1β* expressing neutrophils into the injury site, which negatively influences axonal regeneration in larval zebrafish 8. We analyzed the neutrophil response using immunohistochemistry for Mpx in the *il-1β*:GFP line (labelling *il-1β* expressing neutrophils). Thus we could show that at 2 hours post-lesion (hpl), the peak of the neutrophil response, Cimetidine treatment reduced the overall number of neutrophils (39% reduction in the number of *mpx*:GFP+ neutrophils; Figure 2A-C) and the number of* il-1β* expressing neutrophils (38% reduction in Mpx+/*il-1β*:GFP+ neutrophils; Figure 2A-C). No significant changes were seen at 24 hpl, when neutrophil numbers were greatly reduced, also in controls (Figure 2A,D,E). Counting cell numbers in the *mpx:*GFP reporter line confirmed reduced neutrophil numbers at 2, but not 24 hpl (Figure S2A-C).

Macrophages (*mpeg1*:GFP+), the other major immune cell type in the injury site, did not show differences in overall number (Figure S3A-C) and number expressing *il-1β* (Figure S3D,E,G) at both time points after Cimetidine treatment. However, the GFP signal of the *il-β* transgene reporter showed a non-significant trend to reduced intensity in *mpeg1*:GFP^+^ cells at 24 hpl (Figure S3H), but not at 2 hpl (Figure S3F). These observations further strengthen the notion of an anti-inflammatory action of Cimetidine.

### Cimetidine improves recovery in regeneration-deficient mutant zebrafish with prolonged inflammation

Next, we argued that Cimetidine should be able to improve regenerative outcome in a model of prolonged inflammation in zebrafish. A mutant for the macrophage-determining transcription factor *irf8* lacks macrophages and microglia, which leads to prolonged high numbers of neutrophils and increased levels of *il-1β* expression in the injury site. *Irf8* mutant zebrafish larvae show a reduced anatomical and functional recovery after injury, even after extended post-lesion periods 8.

**Figure 3:**
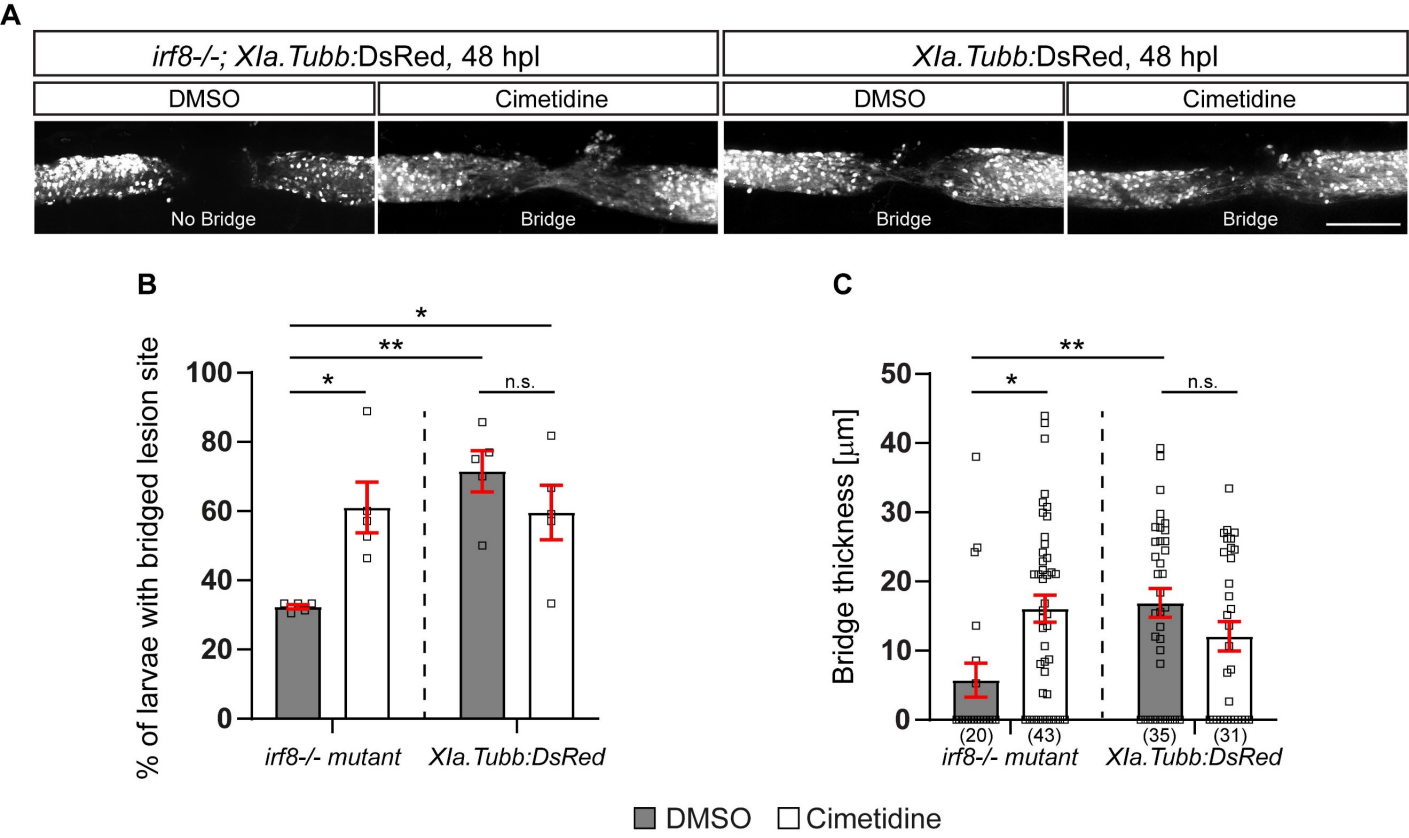
** Cimetidine partially rescues impaired axonal regeneration in *irf8* mutant zebrafish. A:** Representative images of the lateral view of the injury site in cimetidine- and DMSO-treated larvae are shown. An axonal connection across the injury is visualized with DsRed, expressed under a neuronal promoter. Presence of an axonal bridge is indicated in the images. **B:** Quantification by scoring of animals for the presence of an axonal bridge indicates that Cimetidine increases the proportion of *irf8* mutant larvae with an axonal bridge (two-way ANOVA, interaction effect F(1, 16) = 10.86, p* = 0.0046; Tukey's multiple comparison test, *irf8* mutant, DMSO vs *irf8* mutant, cimetidine p* = 0.0215, *irf8*, DMSO vs wt, DMSO p** = 0.0019, *irf8*, DMSO vs wt, cimetidine p* = 0.0295), but does not affect wild-type regeneration. **C:** Measuring the thickness of the axonal bridge yielded similar results for the experimental groups (two-way ANOVA, interaction effect F(1, 125) = 11.60, p*** = 0.0009; Tukey's multiple comparison test, *irf8* mutant, DMSO vs *irf8* mutant, cimetidine p* = 0.0112, *irf8* mutant, DMSO vs wt, DMSO p** = 0.0074). Scale bar: 100 µm

To assess anatomical recovery after injury, we crossed the *Xia.Tubb*:DsRed transgenic reporter line, which labels all neuronal tissue, into the *irf8* mutant line. Regenerative success was determined by scoring larvae as having a “bridge” of fluorescently labelled axons across the injury site. This score has been previously established and correlates with other anatomical measures (thickness of axonal bridge) and functional recovery 32. Using this method, we reproduced the previously reported reduced anatomical recovery in *irf8* mutants compared to wild-type larvae. Incubation with Cimetidine significantly increased the proportion of *irf8* mutants with bridged injury sites and had no significant effect on wild-type animals (Figure 3A,B). The partial rescue of axonal regeneration by Cimetidine in *irf8* mutant larvae was confirmed by measuring the thickness of the axonal bridge (Figure 3C). This shows that Cimetidine can improve axonal regeneration in a mutant with prolonged inflammation and is not toxic to regeneration in wild-type animals.

### Cimetidine acts via hrh2b

Cimetidine has been described as an antagonist of the HRH2 receptor. In the zebrafish CNS, histaminergic innervation is wide-spread with cell bodies mainly located in the posterior hypothalamus 40. Hrh2 receptor binding has also been reported in the zebrafish CNS 41 and intraventricular injection of histamine increases immune cell accumulation and impairs recovery after spinal cord injury in adult zebrafish 42.

**Figure 4:**
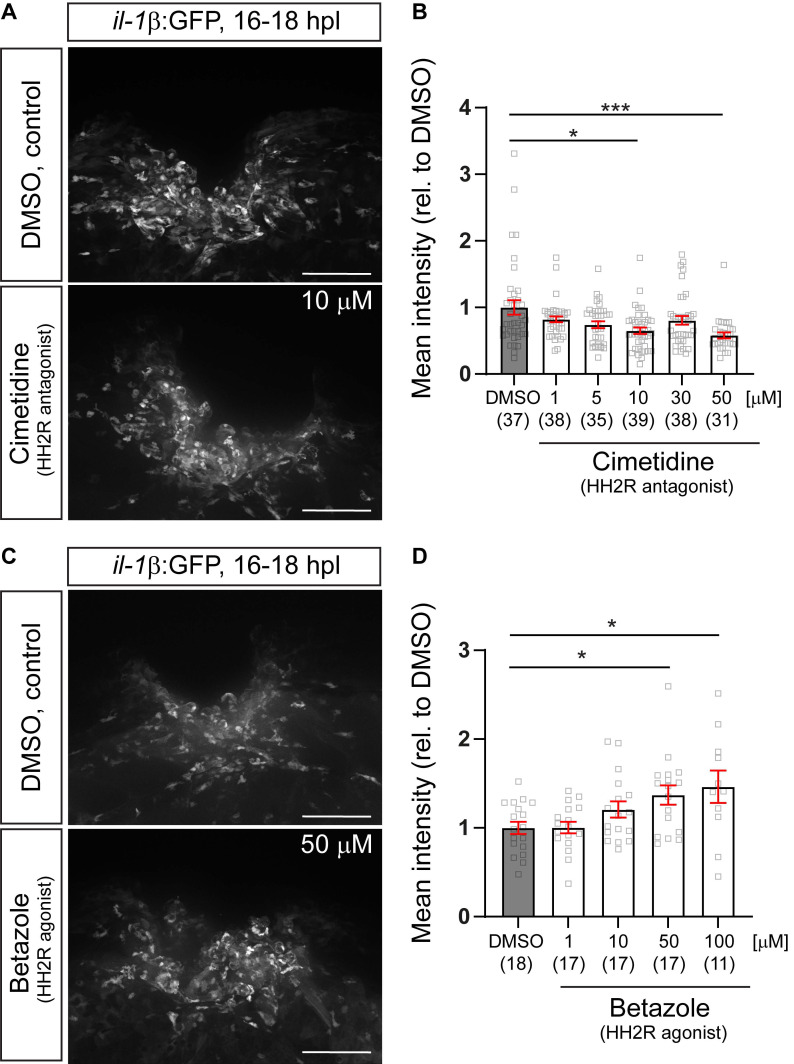
** Pharmacological manipulations HH2R receptor activity indicates positive regulation of *il-1β* expression by the receptors. A:** Representative images of the injury site in *il-1β*:GFP line treated with 10µM cimetidine, or DMSO are shown. **B:** Quantification of the overall GFP fluorescence relative to DMSO-treated larvae indicates a dose-dependent reduction of GFP fluorescence by Cimetidine treatment (Kruskal-Wallis test with Dunn's multiple comparison test, p*** = 0.0003; DMSO vs 10 µM cimetidine, p* = 0.0250; DMSO vs 50 µM cimetidine, p*** = 0.0008). **C:** Representative images of the injury site in *il-1β*:GFP line treated with 50 µM Betazole, or DMSO are shown. **D:** Quantification of the overall GFP fluorescence relative to DMSO-treated larvae reveals a dose-dependent increase of GFP fluorescence by Betazole treatment (Kruskal-Wallis test with Dunn's multiple comparison test, p* = 0.0145, DMSO vs 50 µM Betazole, p* = 0.0422; DMSO vs 100 µM Betazole, p* = 0.0473). Scale bars: 100 µm.

To determine whether the mechanism-of-action of Cimetidine observed is this study is mediated via on-target modulation HRH2 receptor signaling, we first analyzed dose-effect relationships between Cimetidine and GFP fluorescence in the *il-1β*:GFP reporter fish, as well as between the specific HRH2 agonist Betazole and GFP fluorescence. As expected, Cimetidine treatment resulted in a dose-dependent reduction in GFP fluorescence (Figure 4A,B), whereas Betazole led to a dose-dependent increase in GFP fluorescence (Figure 4C,D). This indicated a positive action of Hrh2 receptors on *il-1β* expression levels after spinal injury.

**Figure 5:**
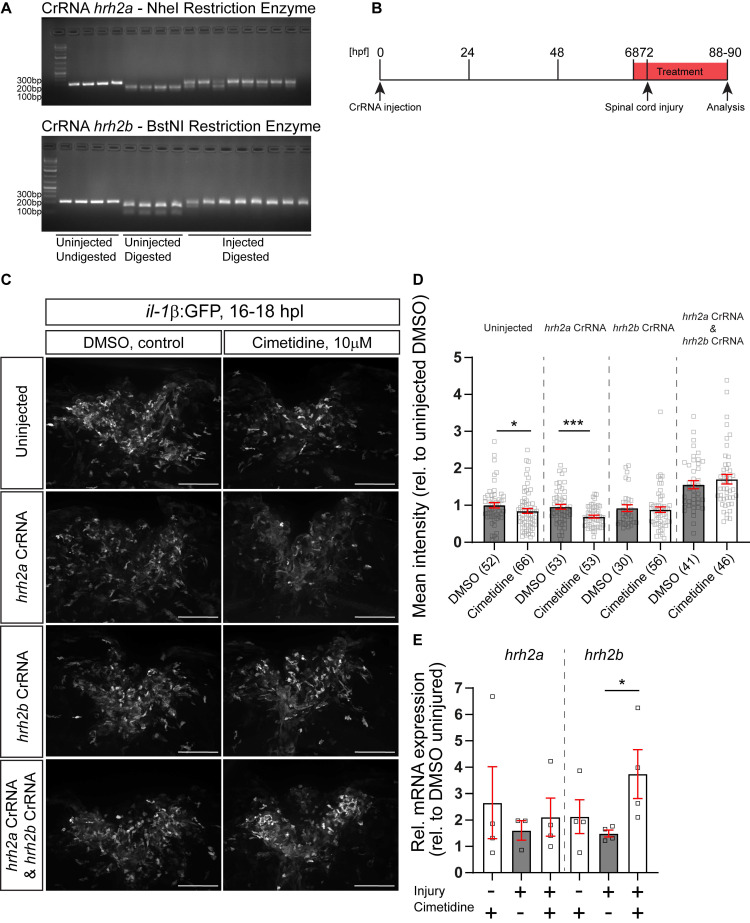
** Somatic receptor mutation indicates that Cimetidine acts through *hrh2b* in zebrafish. A:** Examples of gels used to assess the efficiency of injected CrRNA by RFLP are shown. Each lane represents one embryo. In uninjected controls, a complete band shift is observed, indicating activity of the indicated restriction enzyme, whereas in 8 haCR-injected animals almost no digest occurs, indicating successful mutation of the recognition site. **B:** A schematic timeline of the experimental design, combining somatic mutation with drug treatment is depicted. **C:** Lateral view of the spinal cord injury sites in somatic mutant larvae with and without Cimetidine treatment are shown. **D:** Quantification of the overall GFP fluorescence of the cimetidine-treated somatic receptor mutants relative to DMSO-treated control indicate that in condition in which *hrh2b* haCRs were injected the effect of Cimetidine is abolished. (uninjected condition, Mann-Whitney tests, p* = 0.0373; *hrh2a* CrRNA condition, t-test, p*** = 0.0005). **E:** Cimetidine treatment leads to an increase in the relative *hrh2b* mRNA expression in lesioned wild-type larvae (50 treated or control larvae were pooled per measurement; Kruskal-Wallis test, p* = 0.0242 with Dunn's multiple comparisons test: injured, DMSO vs injured, cimetidine p* = 0.0324). *hrh2a* mRNA expression is unaffected by cimetidine treatment. Scale bars: 100µm.

The zebrafish possesses two genes coding for the Hrh2 receptor, termed *hrh2a* and *hrh2b*. Such paralogs in zebrafish often share the function of the ancestral gene in a complementary fashion 43. To determine which of these genes was responsible for the action of Cimetidine on *il-1β* expression levels, we generated somatic mutants for either receptor using injection of highly-active synthetic RNA Oligo CRISPR guideRNAs (haCRs) into the zygote 14. Targeting restriction enzyme sites in the predicted transmembrane region (*hrh2a*, TM2) or adjacent to the transmembrane domain (*hrh2b*, immediately upstream of TM4) of the genes allowed us to determine efficiency of the haCRs by RFLP. This showed that restriction enzyme digest was strongly inhibited for both genes, indicating quantitative disruption of the genes (Figure 5A).

Next we tested cimetidine on somatic mutants for *hrh2a* and *hrh2b*, generated on the background of the* il-1β*:GFP reporter line, after spinal injury. Somatic mutation of *hrh2b* did not affect overall development of larvae (eye size, body length; Figure S4A-F) or recovery of axon bridging (Figure S4G-H). A sensitive test for recovery of swimming behavior (vibration evoked swim distance; Figure S4J,K) also showed unimpaired recovery (Figure S4L,M). Double mutation of *hrh2a and hrh2b* also had no effects on development or recovery of swimming, indicating that *hrh2a* did no compensate for the absence of* hrh2b* (Figure S5A-D). Similarly, blocking Hrh2 receptors with Cimetidine did also not impair recovery of swimming function (Figure S4I).

In animals in which *hrh2a* was targeted, Cimetidine was still able to reduce GFP fluorescence in *il-1β*:GFP reporter fish after injury, similar to control guides. In contrast, in larvae injected with *hrh2b* haCRs, the action of Cimetidine was abolished. Counter-intuitively, co-injection of haCRs for *hrh2* receptors *a* and *b* alone elevated GFP fluorescence. This indicated a complex relationship between H2 signaling and inflammation. Regardless of this shift in baseline GFP expression, Cimetidine lost its activity when both receptors were targeted in the same larvae (Figure 5B,C,D).

Next, we assessed expression levels of *hrh2a* mRNA and *hrh2b* mRNA in the spinal injury site in the presence and absence of Cimetidine by qRT-PCR. We could detect both receptors in control unlesioned and lesioned animals. Following Cimetidine treatment, the relative expression levels of *hrh2b* mRNA, but not of *hrh2a* mRNA, were increased in lesioned, but not unlesioned animals (Figure 5E). In summary, these experiments support dependency of Cimetidine action on *il-1β* expression on the presence of the *hrh2b* receptor.

### Spinal cord injury increases expression of HDC and H2 receptor staining after spinal cord injury in mice

In the mammalian CNS, histaminergic neurons are mainly located in the posterior hypothalamic region in the tuberomammillary nucleus 44. They project axons rostrally into the brain (thalamus, cortex and other regions) and caudally to the brain stem and spinal cord 44, 45. In the spinal cord the innervation is localized mainly to the dorsal horn. Histamine is expressed in immune cells (mast cells, basophils and macrophages) 45.

We detected very weak labeling of histamine decarboxylase (HDC) in the dorsal horn in the spinal cord of uninjured mice. HDC labeling increased markedly in NeuN+ neurons in dorsal horn, 48h after SCI, and this increase was reduced significantly by Cimetidine treatment (Figure S6A,B). H2 receptor (HRH2) staining was minimal in the normal mouse spinal cord (as previously reported), but interestingly was significantly increased 48h after SCI in the superficial layers of the dorsal horn and in blood vessels along the ventrolateral region of the spinal cord 500 µm caudal to the lesion center. This increased HRH2 staining was reduced to control levels by Cimetidine treatment (Figure S7A,B).

### Cimetidine improves locomotor recovery after spinal cord injury in mice

**Figure 6:**
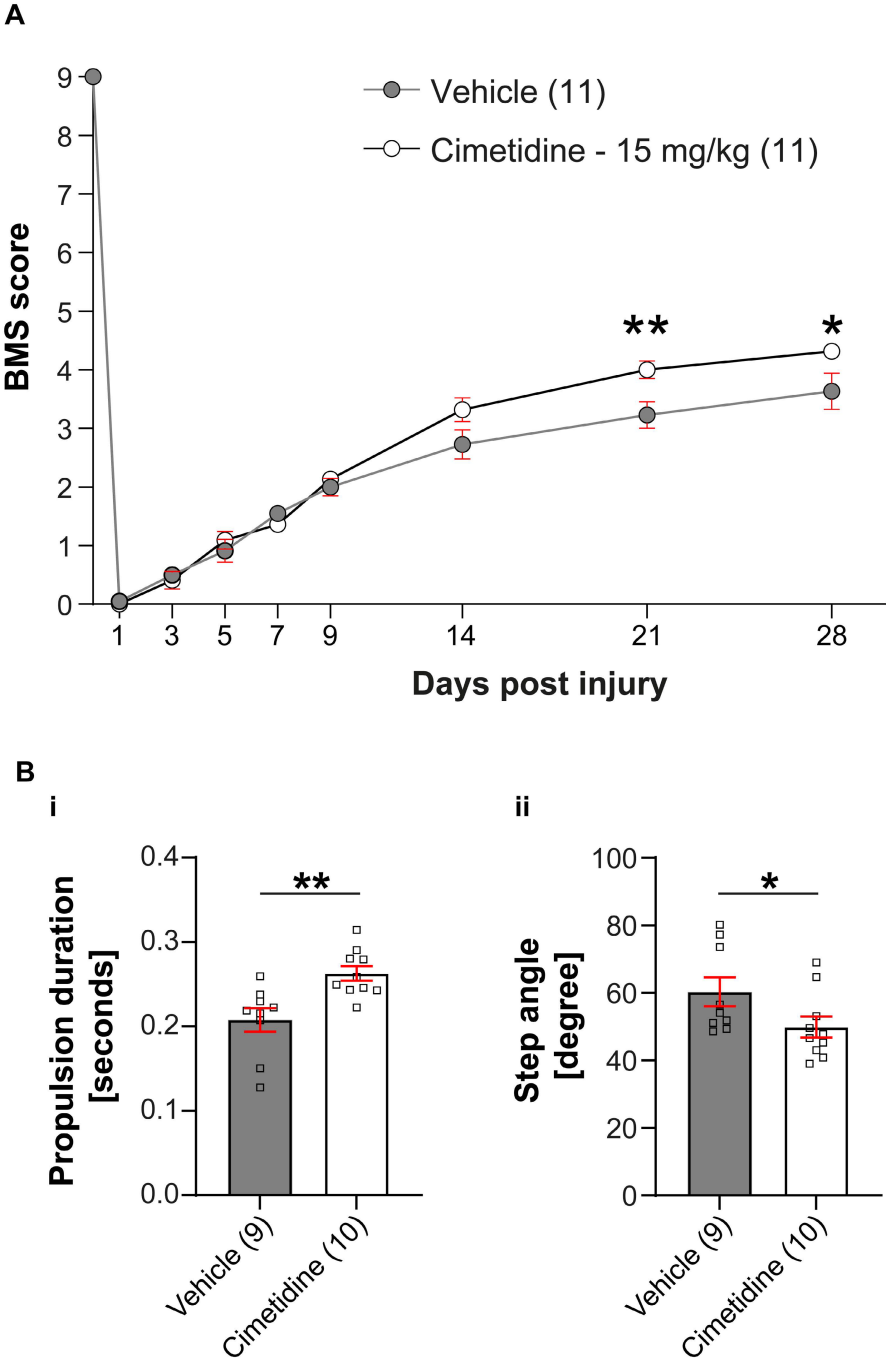
** Cimetidine improves locomotor recovery after spinal injury in mice. A:** Locomotor recovery was assessed using the 9-point Basso Mouse Scale (BMS) analysis. Mice treated with Cimetidine at 15mg/kg under the same conditions showed statistically significant improvement in locomotor control starting at day 21 post-injury (Two-way RM-ANOVA; interaction effect *F*_(8,160)_ = 4.610, *p* < 0.0001; with post-hoc Bonferroni multiple comparison test; *p*_(21d)_ = 0.0058, *p*_(28d)_ = 0.0225, *n* = 11 mice per group). **B:** DigiGait analysis shows significant improvement in propulsion duration (i) and decrease in step angle (ii) with Cimetidine treatment both indicators of improved gait control. (Vehicle vs. Cimetidine: two-tailed Mann Whitney U-test, propulsion duration: p** = 0.0016; step angle: p* = 0.0279; n = 9-10 mice per group).

Given the improved recovery in zebrafish *irf8* mutants, we decided to determine the effect of the drug on recovery in a mouse model of spinal injury. To assess effect of Cimetidine on locomotor recovery, adult female C57BL/6 mice (8-10 weeks of age) were deeply anesthetized and a moderate contusion injury (50 kilodynes force; 400-600 μm tissue displacement) was generated at the T11 thoracic vertebral level using the Infinite Horizon Impactor device (Precision Scientific Instrumentation, Lexington, KY), as previously described 46, 47. Mice were injected intraperitoneally with Cimetidine (15 mg/kg, twice daily) for 14 days, starting immediately after contusion injury. Locomotor recovery was assessed using the 9-point Basso Mouse Scale (BMS) 35. Mice treated with Cimetidine showed significant improvement in locomotor recovery starting at 21 days post-injury (Figure 6A). DigiGait analysis showed that Cimetidine treatment increased propulsion duration (p≤ 0.5) and reduced step angle (p≤ 0.5) as compared to vehicle treated SCI controls. Changes in these two parameters also signify improvement in gait control (Figure 6B).

### Secondary damage after spinal cord injury in mice is reduced by Cimetidine treatment

We next assessed the effects of Cimetidine on various histological measures that may account for the improved locomotor recovery. These comprise assessment of lesion size, myelin loss, neuronal loss, and serotonergic innervation of the ventral horn 1mm caudal to the lesion center at 28 days post-injury.

**Figure 7:**
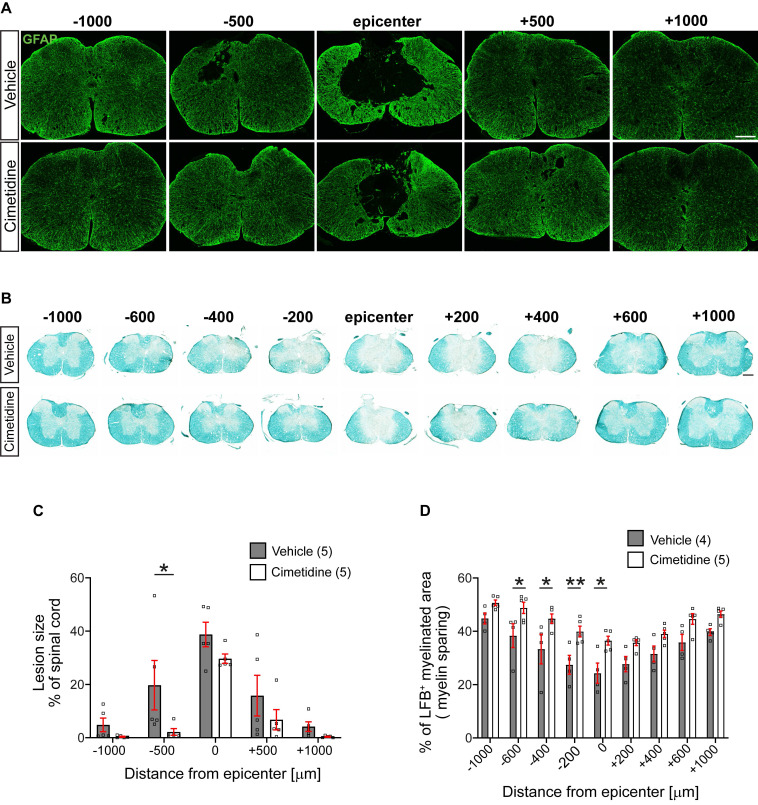
** Treatment with Cimetidine reduces secondary damage induced by spinal cord contusion injury in mice. A:** Confocal images of the spinal cord at 28-day survival after injury stained with anti-GFAP are shown. Note that the size and extent of the lesion is smaller in the Cimetidine treated group compared to the vehicle-treated control (quantified in C). **B:** Luxol fast blue (LFB) stained sections of the spinal cord 28 days after SCI. Note greater sparing of myelin LFB staining in the Cimetidine-treated group (quantified in D). **C:** Quantification of tissue loss is shown. Note although the mean values in the Cimetidine treated group are smaller than in the vehicle-treated group, the value reaches statistical significance only at - 500 µm rostral to the lesion due to the variability between animals (Two-way RM-ANOVA; group effect *F*_(1,8)_ = 3.188, *p* = 0.1120; with Sidak's multiple comparison test; p*_(-500)_ = 0.0372, *n* = 5 mice per group. Note also that the longitudinal extent of the lesion is smaller in the Cimetidine-treated group. **D:** Mice treated with Cimetidine show a significant reduction of myelin loss (LFB staining) from the center of the lesion to 600 μm rostrally (Two-way RM-ANOVA; group effect *F*_(1,7)_ = 12.35, *p* = 0.0098; with post-hoc Bonferroni multiple comparison test; p*_(-600µm)_ = 0.0438, p*_(-400µm)_ = 0.0204, p**_(-200µm)_ = 0.0084, p*_(center)_ = 0.0103, *n* = 4-5 mice per group). Scale bars = 200 µm.

We assessed the size of the lesion from serial tissue sections stained with anti-GFAP antibodies. GFAP labels most finely-branched astrocytic processes present throughout the tissue and thus reveals presence of CNS tissue. Lesion size was estimated as percent of tissue loss per cross section. This analysis showed that in vehicle treated controls, the lesion often extended the entire 2 mm length of spinal cord examined (i.e., from -1000 µm to +1000 µm rostral and caudal to the lesion center). In contrast, Cimetidine treatment reduced the rostro-caudal length of the lesion by ~50%, i.e., extended from - 500µm rostral to + 500µm caudal to the center (Figure 7A). The area of the lesion in cross-sections was also smaller in the Cimetidine treated group as compared to vehicle treated controls at all distances. However, due to variability between animals, these results reached statistical significance only at 500 µm rostrally (Figure 7C).

Myelin loss is another indicator of secondary damage, which was assessed using Luxol fast blue (LFB) staining (Figure 7B). This tissue stain was quantified as a percentage of the whole cross-section of the spinal cord. Cimetidine treatment increased myelin sparing as compared with vehicle-treated control mice. The mean values showed differences along the entire 2 mm length examined but reached statistical significance in the region rostral to the lesion center (Figure 7D).

**Figure 8:**
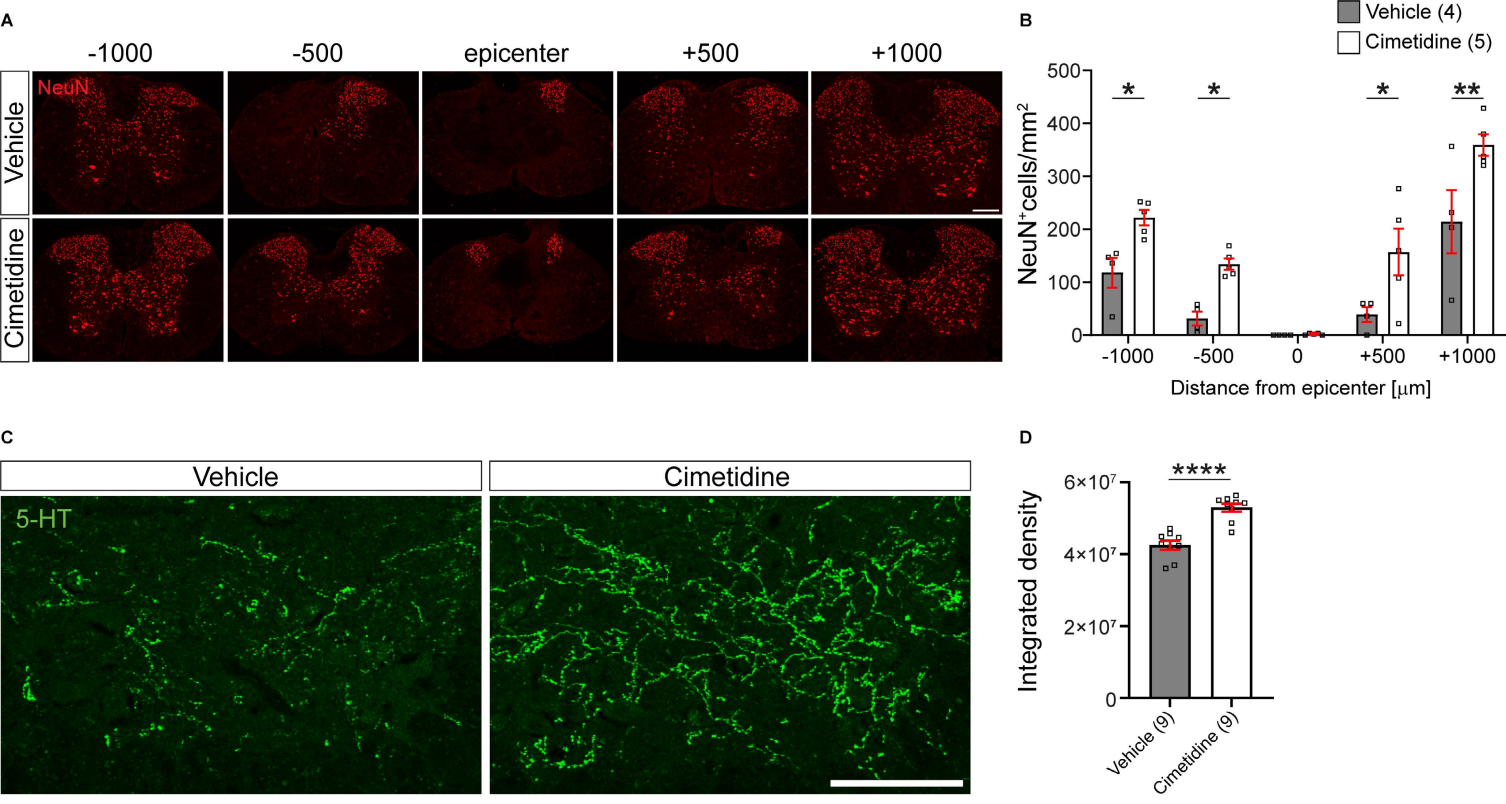
** Cimetidine treatment leads to higher density of neurons and greater serotonergic innervation after spinal cord injury in mice. A:** Single plane confocal images of spinal cord cross-sections 28 days after spinal cord contusion injury stained with anti-NeuN antibody to label neurons. Note the greater sparing of neurons in the Cimetidine-treated animal compared to the vehicle group. **B:** Quantification of NeuN labeling in the ventral horn is shown. Mice treated with Cimetidine showed increased survival of NeuN+ neurons in the ventral horn of the spinal cord as compared to controls on either side of the lesion center (Two-way RM-ANOVA; group effect *F*_(1,7)_ = 20.65, *p* = 0.0027; with post-hoc Bonferroni multiple comparison test; p*_(-1000µm)_ = 0.0448, p*_(-500µm)_ = 0.0496, p*_(+500µm)_ = 0.0172, p**_(+1000µm)_ = 0.0024, *n* = 4-5 mice per group.)** C:** Increased serotonergic innervation of the ventral horn treated with Cimetidine is observed at 1mm caudal to the lesion center by 5-HT immunohistochemistry.** D:** Quantification by optical density measurements shows that there is significantly greater 5-HT innervation in Cimetidine treated mice than in vehicle-treated controls. Mann-Whitney unpaired t test, *p* ≤ 0.0001, n = 9 per group*;* Scale bar = 200 µm and 100 µm for A and C, respectively.

In line with better preservation of tissue and myelin by Cimetidine treatment, we also found a higher density of NeuN immunolabelled neurons in the ventral horn of the spinal cord compared to vehicle-treated controls (Figure 8A). This was quantified by counting NeuN+ cell profiles in single optical sections and indicated that Cimetidine treatment increased neuronal survival. At 500 µm rostral and caudal to the lesion center, we observed a 4-fold higher number of neuronal profiles than in vehicle treated controls and at 1000 µm rostral and caudal to the center the increase was still ~ 70 % - 90 % (Figure 8B).

In addition, we also assessed the density of serotonergic (5-HT) innervation of the ventral horn region 1 mm caudal to the injury center in cross-sections of the spinal cord (Figure 8C). The descending serotonergic input from the raphe nuclei in the brainstem to the ventral horn neurons is important for locomotor control 48, 49. Cimetidine treated mice showed a significantly greater density of serotonergic innervation of the ventral horn grey matter caudal to the injury site than control injured mice (Figure 8D). This was likely due to greater sparing and/or sprouting of serotonergic fibers. Hence, all histological parameters tested showed significantly improved spinal tissue preservation by Cimetidine treatment.

### Cimetidine changes the inflammatory environment in injured spinal cord of mice

**Figure 9:**
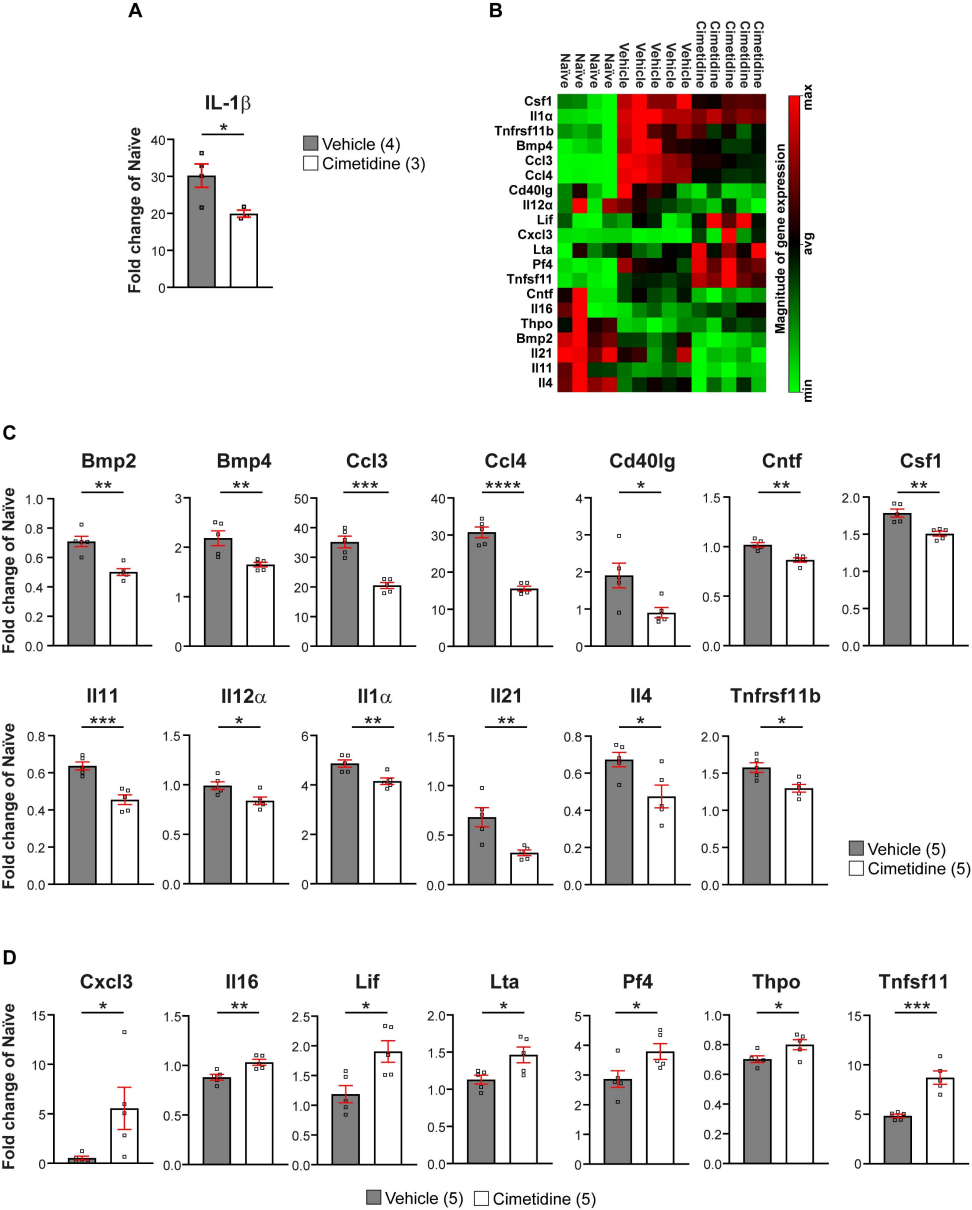
** Cimetidine changes the cytokine profile of the injury site in mice. A:** qRT-PCR shows significant decrease in* il-1β* mRNA expression after 1 day of Cimetidine treatment (one-way ANOVA with Tukey's multiple comparison test, p**** < 0.0001, vehicle vs. cimetidine p* = 0.0219). **B:** Results of the RT^2^ profiler cytokine array (PAMM-150Z) after 10 days of Cimetidine treatment depicted as a heat map indicating genes with decreased (green) and increased (red) transcript levels (quantification shown in C and D) in individual mice in the three indicated groups is shown. **C:** Quantification of genes with decreased transcript levels is shown for B. **D**: Quantification of genes with increased transcript levels is shown for B. Statistics for C,D: vehicle vs. cimetidine: two-tailed Mann Whitney U-test for all comparisons; *Bmp2*: p** = 0.0012; *Bmp4*: p** = 0.009; *Ccl3*: p*** = 0.0002; *Ccl4*: p**** ≤ 0.0001; *Cd40lg*: p* = 0.0238; *Cntf*: p** = 0.0013; *Csf1*: p** = 0.0023; *Il11*: p*** = 0.0007; *Il12α*: p* = 0.0219; *Il1α*: p** = 0.0069; *Il21*: p** = 0.0073; *Il4*: p* = 0.0257; *Tnfrsf11b*: p* = 0.0104; *Cxcl3*: p* = 0.0469; *Il16*: p** = 0.0065; *Lif*: p* = 0.0148; *Lta*: p* = 0.0264; *Pf4*: p* = 0.0418; Thpo: p* = 0.0425; *Tnfsf11*: p*** = 0.0005; n = 4-5 mice per group.

As IL-1β expression changes rapidly after SCI in mice 50, we treated injured mice for 1 day with Cimetidine (15mg/kg, twice) before taking a 3 mm length of the spinal cord to assess *il-1β* mRNA levels by qRT-PCR. This short treatment with Cimetidine significantly reduced *il-1β* expression (Figure 9A) as in zebrafish. We also assessed changes in IL-1β expression at the protein level by immunofluorescence staining. For this, a 48h treatment with Cimetidine (15 mg/kg, daily) was used. Immunolabelling for IL-1β was barely detectable in the uninjured spinal cord but increased significantly 48h after SCI with labeling localized to GFAP+ astrocytes in the ventral and lateral region of the injured spinal cord (Figure S8A). Importantly, this increase in IL-1β was significantly reduced by Cimetidine treatment (Figure S8B).

We next assessed whether longer treatment with Cimetidine for 10 days post-SCI would modulate the immune reaction more broadly after injury in mice, as seen in zebrafish larvae. For this, we screened 84 inflammation-related genes using the RT^2^ profiler cytokine array (PAMM-150Z) (Qiagen). A 3 mm length of spinal cord tissue centered on the injury site was taken after 10 days of Cimetidine treatment (15mg/kg), which was started immediately after contusion injury. Of the 84 inflammation associated genes screened, expression of 20 were altered by Cimetidine treatment. These included 13 that were downregulated (Figure 9B,C) and 7 that were upregulated (Figure 9B,D), compared to vehicle treated controls. Heat map of the rest of the 64 unchanged genes is shown in Figure S9. The most substantially downregulated genes include the chemokine CCL3 (macrophage inflammatory protein 1α), which is involved in the recruitment and activation of neutrophils after SCI 51; and CCL4 (macrophage inflammatory protein 1β) involved in the recruitment of monocyte and other immune cells. The substantially upregulated genes include Pf4 (CXCL4) known to be involved in wound repair 52, and thus may be beneficial after SCI; and Tfnsf11 (RANKL) which influences macrophage differentiation 53, 54. Overall, these findings suggest that Cimetidine changes the inflammatory response after SCI to be more pro-regenerative.

We next assessed if Cimetidine treatment affected inflammatory cell infiltration and activation, in particular changes in CD11b+ macrophages/microglia. We found that Cimetidine treatment reduces the number of CD11b+ macrophages/microglia and extent of CD11b staining at the lesion site at 4 weeks after SCI (Figure S10A,B). Furthermore, Cimetidine treatment reduces lipid peroxidation in the lesion epicenter as detected by 4HNE (4-Hydroxynonenal) staining, compared to vehicle treated SCI controls (Figure S11A,B). These results provide further evidence that Cimetidine reduces inflammation and lipid peroxidation after SCI in mice.

## Discussion

Through a drug screening assay in larval zebrafish, we have identified the histamine H2 receptor antagonist Cimetidine as an immuno-modulator after spinal cord injury. Cimetidine modulates the immune response in both zebrafish and mice and promotes recovery in a zebrafish model of prolonged inflammation and in adult mice after spinal cord injury. Increased functional recovery in mice is accompanied by more extensive neural tissue preservation.

Improved behavioral recovery in mice on the BMS scale correlated with superior tissue sparing. The lesion size was reduced, and more neurons and myelinated axons were spared by Cimetidine treatment. Functionally important descending 5-HT fibers showed a higher density of innervation of the ventral horn caudal to the injury likely due to better preservation, sprouting or regrowth. Previous short reports showed that pre-treatment with Cimetidine before spinal injury improved spinal conductivity, edema formation and neuronal nitric oxide synthase in the hours after injury 55-57. We now show here that a translationally relevant post-injury treatment with Cimetidine leads to long-term behavioral improvements, most likely by modulating the cytokine environment in the lesion area.

Immuno-modulation by Cimetidine in mice is demonstrated by our observation that genes involved in recruitment of immune cells, such as Il-1β, CCL3, CCL4 were robustly reduced in expression. At the same time, immune system differentiation factors, such as Pf4 and Tnfsf11 showed increased detectability after injury. The decreased and increased detectability of specific immune system related genes indicates that Cimetidine treatment genuinely modulates the immune system. This is perhaps in contrast to the current only treatment option, Methylprednisolone, which generally suppresses the immune response, and therefore, potentially both the beneficial functions of the immune reaction, as well as detrimental cytotoxic effects of chronic inflammation 5.

Several lines of evidence suggest that reduced abundance of CCL3 and CCL4 expression after Cimetidine treatment is pro-regenerative. Firstly, CCL3 deficient mice show improved recovery after spinal injury 51. Secondly, targeting the immune system with infusion of exogenous Maresin 1 after spinal injury in female mice also leads to reduced expression of CCL3 and CCL4 and improved tissue preservation and recovery 58. Interestingly, both CCL3 and CCL4 can signal through the same receptor, CCR5 59, and one of the factors we found to be increased in expression after injury, Pf4, can down-regulate this receptor in human monocytes 52. This leads us to speculate that the CCL4/CCL5/CCR5 axis is a possible target of the Cimetidine intervention. However, future research will have to show how exactly this altered cytokine profile acts on different cell types in the injury site that influence regenerative success, such as immune cells, fibroblasts, astrocytes, oligodendrocyte precursor cells and the matrix they produce 60-62.

Levels of the natural HRH2 ligand histamine have been shown to be increased after spinal injury in mammals 63-65 and histamine is mostly discussed in the context of pain perception. These functions are mediated mostly by HRH3 and HRH4 receptors, such that the HRH2 antagonist Cimetidine may only interfere minimally with pain or neuropathic pain after spinal injury 66.

We have identified Cimetidine in a screening approach in larval zebrafish and we have collected evidence that Cimetidine's action is likely “on target” through the HRH2 receptor in the fish model. This is supported by our observation that when we introduce a somatic mutation of the *hrh2b* gene the action of Cimetidine is abolished. Moreover, an agonist of the receptor has the opposite effect on *il-1β* expression. We also show a positive feedback mechanism, by which Cimetidine treatment upregulated receptor mRNA expression specifically. This is a known action of Cimetidine in mammals 67, indicating that mechanisms of action in zebrafish may be similar to those in mammals and target the HRH2 receptor. However, here we detected that at the protein level there was a reduction of HRH2 expression in adult mice, suggesting more complex regulation mechanisms. The observation that Cimetidine has an overall similar mode of action in larval zebrafish as has been previously well characterized in mammals, validates the zebrafish screening approach for drugs that are functional in mammals.

Of note, we present evidence for different cell types to show a reaction to Cimetidine. For example, neutrophils in zebrafish larvae are reduced in number and expression of *il-1β,* and further, detectability of Il-1β is reduced in astrocytes in mice. Macrophages are reduced in number (mice) or show a tendency towards reduced expression of *il-1β* (zebrafish). These observations could reflect biological differences between larval zebrafish and adult mice or be a consequence of different stages of the dynamic immune response being assayed with species-specific tools. However, parameters in both species invariably show that Cimetidine reduces inflammation.

The complex neuro-immune interactions we observe on zebrafish would be difficult to model in an alternative cell culture or animal model to screen in a cost-effective way. Nevertheless, one limitation of the system is that larval zebrafish do not possess an adaptive immune system at the age investigated 68, such that actions on the adaptive immune system could not have been detected in our screen, potentially leading to false-negative results. However, the innate immune system is reacting to an injury first also in adult mammals in a stereotypical and conserved sequence that is similar to that in larval zebrafish 8. Adaptive immune cells only enter the injury site several days after injury in mammals 69. Hence, actions on early innate immunity that are detected in larval zebrafish are likely to also apply to the initial reaction of the innate immune system in mammals.

False-negative results could also result from the design of the study, which starts out with a visual inspection of the larvae to determine potentially decreased reporter gene fluorescence. This could be standardised in future by automation, using automated handling of larvae and image analysis 17, 70. False-positive results could likewise be minimised by automation. However, due to several validation steps, we were able to eliminate false-positive results efficiently, such that the screen yielded a “hit rate” that was in line with similar screens in zebrafish 71, 72.

Overall, our results suggest that HRH2 inhibition could be a treatment option for spinal cord injury. There are a number of preclinical studies, in which the immune system is modulated, and functional improvement is achieved. This may be either by direct application of immune signaling molecules 73-76, or transplantation of mesenchymal 77, 78 or neural stem cells 79, which by themselves modulate the immune response 5. Here we show that a non-invasive systemic treatment with an FDA-approved drug with an excellent safety record 80 can lead to functionally relevant modulation of the immune response in mice. Efficacy and potential side-effects have to be weighed against those of other FDA-approved potentially immunomodulatory drugs found to be effective in improving behavior outcomes in pre-clinical spinal injury studies 81, 82.

One aspect that might be of importance in the future to enhance cimetidine action could be timing of the treatment, as the immune response is highly dynamic, with some potential benefits of early pro-inflammatory gene expression, such that a delayed treatment could be more effective 5, 83. Other HRH2 antagonists could also be tested for their potentially higher efficacy 84. Finally, potential synergies with other drug treatments that do not primarily target immune system-related inhibitory actions in the spinal cord environment, such as those of myelin 85, 86, scar-associated proteoglycans 87, 88 or the fibrotic scar 89, should be explored.

## Supplementary Material

Supplementary figures and table.

## References

[1] Dumont RJ, Okonkwo DO, Verma S, Hurlbert RJ, Boulos PT, Ellegala DB (2001). Acute spinal cord injury, part I: pathophysiologic mechanisms. Clin Neuropharmacol.

[2] McDonald JW, Sadowsky C (2002). Spinal-cord injury. Lancet.

[3] Sekhon LH, Fehlings MG (2001). Epidemiology, demographics, and pathophysiology of acute spinal cord injury. Spine (Phila Pa 1976).

[4] Greenhalgh AD, David S, Bennett FC (2020). Immune cell regulation of glia during CNS injury and disease. Nat Rev Neurosci.

[5] Chio JCT, Xu KJ, Popovich P, David S, Fehlings MG (2021). Neuroimmunological therapies for treating spinal cord injury: Evidence and future perspectives. Exp Neurol.

[6] Boato F, Rosenberger K, Nelissen S, Geboes L, Peters EM, Nitsch R (2013). Absence of IL-1beta positively affects neurological outcome, lesion development and axonal plasticity after spinal cord injury. J Neuroinflammation.

[7] Kroner A, Greenhalgh AD, Zarruk JG, Passos Dos Santos R, Gaestel M, David S (2014). TNF and increased intracellular iron alter macrophage polarization to a detrimental M1 phenotype in the injured spinal cord. Neuron.

[8] Tsarouchas TM, Wehner D, Cavone L, Munir T, Keatinge M, Lambertus M (2018). Dynamic control of proinflammatory cytokines Il-1β and Tnf-α by macrophages in zebrafish spinal cord regeneration. Nat Commun.

[9] Paolicelli RC, Sierra A, Stevens B, Tremblay ME, Aguzzi A, Ajami B (2022). Microglia states and nomenclature: A field at its crossroads. Neuron.

[10] Fehlings MG, Wilson JR, Tetreault LA, Aarabi B, Anderson P, Arnold PM (2017). A Clinical Practice Guideline for the Management of Patients With Acute Spinal Cord Injury: Recommendations on the Use of Methylprednisolone Sodium Succinate. Global Spine J.

[11] Fehlings MG, Wilson JR, Harrop JS, Kwon BK, Tetreault LA, Arnold PM (2017). Efficacy and Safety of Methylprednisolone Sodium Succinate in Acute Spinal Cord Injury: A Systematic Review. Global Spine J.

[12] Lam PY, Peterson RT (2019). Developing zebrafish disease models for *in vivo* small molecule screens. Curr Opin Chem Biol.

[13] Patton EE, Zon LI, Langenau DM (2021). Zebrafish disease models in drug discovery: from preclinical modelling to clinical trials. Nat Rev Drug Discov.

[14] Keatinge M, Tsarouchas TM, Munir T, Porter NJ, Larraz J, Gianni D (2021). CRISPR gRNA phenotypic screening in zebrafish reveals pro-regenerative genes in spinal cord injury. PLoS Genet.

[15] Chapela D, Sousa S, Martins I, Cristovao AM, Pinto P, Corte-Real S (2019). A zebrafish drug screening platform boosts the discovery of novel therapeutics for spinal cord injury in mammals. Sci Rep.

[16] Klatt Shaw D, Mokalled MH Efficient CRISPR/Cas9 mutagenesis for neurobehavioral screening in adult zebrafish. G3 (Bethesda). 2021: jkab089.

[17] Oprişoreanu AM (2022). Perspective on automated *in vivo* drug screening using the chodl mutant zebrafish line. Neural Regen Res.

[18] Pott A, Rottbauer W, Just S (2020). Streamlining drug discovery assays for cardiovascular disease using zebrafish. Expert Opin Drug Discov.

[19] Becker T, Becker CG (2014). Axonal regeneration in zebrafish. Curr Opin Neurobiol.

[20] Alper SR, Dorsky RI (2022). Unique advantages of zebrafish larvae as a model for spinal cord regeneration. Front Mol Neurosci.

[21] Sommer F, Torraca V, Meijer AH (2020). Chemokine Receptors and Phagocyte Biology in Zebrafish. Front Immunol.

[22] Andries L, De Groef L, Moons L (2020). Neuroinflammation and Optic Nerve Regeneration: Where Do We Stand in Elucidating Underlying Cellular and Molecular Players?. Curr Eye Res.

[23] Cavone L, McCann T, Drake LK, Aguzzi EA, Oprişoreanu AM, Pedersen E (2021). A unique macrophage subpopulation signals directly to progenitor cells to promote regenerative neurogenesis in the zebrafish spinal cord. Dev Cell.

[24] Shiau CE, Kaufman Z, Meireles AM, Talbot WS (2015). Differential Requirement for irf8 in Formation of Embryonic and Adult Macrophages in Zebrafish. PLoS One.

[25] Beck KD, Nguyen HX, Galvan MD, Salazar DL, Woodruff TM, Anderson AJ (2010). Quantitative analysis of cellular inflammation after traumatic spinal cord injury: evidence for a multiphasic inflammatory response in the acute to chronic environment. Brain.

[26] Hayes KC, Hull TC, Delaney GA, Potter PJ, Sequeira KA, Campbell K (2002). Elevated serum titers of proinflammatory cytokines and CNS autoantibodies in patients with chronic spinal cord injury. J Neurotrauma.

[27] Ellett F, Lieschke GJ (2010). Zebrafish as a model for vertebrate hematopoiesis. Curr Opin Pharmacol.

[28] Ogryzko NV, Lewis A, Wilson HL, Meijer AH, Renshaw SA, Elks PM (2019). Hif-1α-Induced Expression of Il-1β Protects against Mycobacterial Infection in Zebrafish. J Immunol.

[29] Peri F, Nusslein-Volhard C (2008). Live imaging of neuronal degradation by microglia reveals a role for v0-ATPase a1 in phagosomal fusion *in vivo*. Cell.

[30] Ellett F, Pase L, Hayman JW, Andrianopoulos A, Lieschke GJ (2011). mpeg1 promoter transgenes direct macrophage-lineage expression in zebrafish. Blood.

[31] Ohnmacht J, Yang Y, Maurer GW, Barreiro-Iglesias A, Tsarouchas TM, Wehner D (2016). Spinal motor neurons are regenerated after mechanical lesion and genetic ablation in larval zebrafish. Development.

[32] Wehner D, Tsarouchas TM, Michael A, Haase C, Weidinger G, Reimer MM (2017). Wnt signaling controls pro-regenerative Collagen XII in functional spinal cord regeneration in zebrafish. Nat Commun.

[33] Norton WH, Stumpenhorst K, Faus-Kessler T, Folchert A, Rohner N, Harris MP (2011). Modulation of Fgfr1a signaling in zebrafish reveals a genetic basis for the aggression-boldness syndrome. J Neurosci.

[34] Ibarra-Garcia-Padilla R, Howard AGAt, Singleton EW, Uribe RA (2021). A protocol for whole-mount immuno-coupled hybridization chain reaction (WICHCR) in zebrafish embryos and larvae. STAR Protoc.

[35] Basso DM, Fisher LC, Anderson AJ, Jakeman LB, McTigue DM, Popovich PG (2006). Basso Mouse Scale for locomotion detects differences in recovery after spinal cord injury in five common mouse strains. J Neurotrauma.

[36] Livak KJ, Schmittgen TD (2001). Analysis of relative gene expression data using real-time quantitative PCR and the 2(-Delta Delta C(T)) Method. Methods.

[37] Grisold W, Cavaletti G, Windebank AJ (2012). Peripheral neuropathies from chemotherapeutics and targeted agents: diagnosis, treatment, and prevention. Neuro Oncol.

[38] Carozzi VA, Canta A, Oggioni N, Sala B, Chiorazzi A, Meregalli C (2010). Neurophysiological and neuropathological characterization of new murine models of chemotherapy-induced chronic peripheral neuropathies. Exp Neurol.

[39] Carozzi VA, Renn CL, Bardini M, Fazio G, Chiorazzi A, Meregalli C (2013). Bortezomib-induced painful peripheral neuropathy: an electrophysiological, behavioral, morphological and mechanistic study in the mouse. PLoS One.

[40] Kaslin J, Nystedt JM, Ostergard M, Peitsaro N, Panula P (2004). The orexin/hypocretin system in zebrafish is connected to the aminergic and cholinergic systems. J Neurosci.

[41] Peitsaro N, Sundvik M, Anichtchik OV, Kaslin J, Panula P (2007). Identification of zebrafish histamine H1, H2 and H3 receptors and effects of histaminergic ligands on behavior. Biochem Pharmacol.

[42] Huang SB, Zhao HD, Wang LF, Sun MF, Zhu YL, Wu YB (2017). Intradiencephalon injection of histamine inhibited the recovery of locomotor function of spinal cord injured zebrafish. Biochem Biophys Res Commun.

[43] Cañestro C, Yokoi H, Postlethwait JH (2007). Evolutionary developmental biology and genomics. Nat Rev Genet.

[44] Schwartz JC, Arrang JM, Garbarg M, Pollard H, Ruat M (1991). Histaminergic transmission in the mammalian brain. Physiol Rev.

[45] Watanabe T, Taguchi Y, Shiosaka S, Tanaka J, Kubota H, Terano Y (1984). Distribution of the histaminergic neuron system in the central nervous system of rats; a fluorescent immunohistochemical analysis with histidine decarboxylase as a marker. Brain Res.

[46] Ghasemlou N, Kerr BJ, David S (2005). Tissue displacement and impact force are important contributors to outcome after spinal cord contusion injury. Exp Neurol.

[47] Lopez-Vales R, Ghasemlou N, Redensek A, Kerr BJ, Barbayianni E, Antonopoulou G (2011). Phospholipase A2 superfamily members play divergent roles after spinal cord injury. Faseb J.

[48] Ribotta MG, Provencher J, Feraboli-Lohnherr D, Rossignol S, Privat A, Orsal D (2000). Activation of locomotion in adult chronic spinal rats is achieved by transplantation of embryonic raphe cells reinnervating a precise lumbar level. J Neurosci.

[49] Antri M, Orsal D, Barthe JY (2002). Locomotor recovery in the chronic spinal rat: effects of long-term treatment with a 5-HT2 agonist. The European journal of neuroscience.

[50] Francos-Quijorna I, Amo-Aparicio J, Martinez-Muriana A, López-Vales R (2016). IL-4 drives microglia and macrophages toward a phenotype conducive for tissue repair and functional recovery after spinal cord injury. Glia.

[51] Pelisch N, Rosas Almanza J, Stehlik KE, Aperi BV, Kroner A (2020). CCL3 contributes to secondary damage after spinal cord injury. J Neuroinflammation.

[52] Schwartzkopff F, Petersen F, Grimm TA, Brandt E (2012). CXC chemokine ligand 4 (CXCL4) down-regulates CC chemokine receptor expression on human monocytes. Innate Immun.

[53] Deuell KA, Callegari A, Giachelli CM, Rosenfeld ME, Scatena M (2012). RANKL enhances macrophage paracrine pro-calcific activity in high phosphate-treated smooth muscle cells: dependence on IL-6 and TNF-α. J Vasc Res.

[54] Meng YH, Zhou WJ, Jin LP, Liu LB, Chang KK, Mei J (2017). RANKL-mediated harmonious dialogue between fetus and mother guarantees smooth gestation by inducing decidual M2 macrophage polarization. Cell Death Dis.

[55] Sharma HS, Vannemreddy P, Patnaik R, Patnaik S, Mohanty S (2006). Histamine receptors influence blood-spinal cord barrier permeability, edema formation, and spinal cord blood flow following trauma to the rat spinal cord. Acta Neurochir Suppl.

[56] Winkler T, Sharma HS, Stålberg E, Olsson Y, Nyberg F (1995). Role of histamine in spinal cord evoked potentials and edema following spinal cord injury: experimental observations in the rat. Inflamm Res.

[57] Sharma HS, Patnaik R, Muresanu DF, Lafuente JV, Ozkizilcik A, Tian ZR (2017). Histaminergic Receptors Modulate Spinal Cord Injury-Induced Neuronal Nitric Oxide Synthase Upregulation and Cord Pathology: New Roles of Nanowired Drug Delivery for Neuroprotection. Int Rev Neurobiol.

[58] Francos-Quijorna I, Santos-Nogueira E, Gronert K, Sullivan AB, Kopp MA, Brommer B (2017). Maresin 1 Promotes Inflammatory Resolution, Neuroprotection, and Functional Neurological Recovery After Spinal Cord Injury. J Neurosci.

[59] Mukaida N, Sasaki SI, Baba T (2020). CCL4 Signaling in the Tumor Microenvironment. Adv Exp Med Biol.

[60] Bradbury EJ, Burnside ER (2019). Moving beyond the glial scar for spinal cord repair. Nat Commun.

[61] Courtine G, Sofroniew MV (2019). Spinal cord repair: advances in biology and technology. Nat Med.

[62] Kroner A, Rosas Almanza J (2019). Role of microglia in spinal cord injury. Neurosci Lett.

[63] Panneerselvam K, Cherian L, Kuruvilla A, Theodore DR, Abraham J (1989). Changes in noradrenaline and histamine in monkey spinal cords traumatised by weight drop, compression and subsequent decompression. Indian J Exp Biol.

[64] Naftchi NE, Demeny M, DeCrescito V, Tomasula JJ, Flamm ES, Campbell JB (1974). Biogenic amine concentrations in traumatized spinal cords of cats. Effect of drug therapy. J Neurosurg.

[65] Tseng LH, Chen I, Lin YH, Liang CC, Lloyd LK (2010). Genome-based expression profiling study following spinal cord injury in the rat: An array of 48-gene model. Neurourol Urodyn.

[66] Nuutinen S, Panula P (2010). Histamine in neurotransmission and brain diseases. Adv Exp Med Biol.

[67] Smit MJ, Leurs R, Alewijnse AE, Blauw J, Van Nieuw Amerongen GP, Van De Vrede Y (1996). Inverse agonism of histamine H2 antagonist accounts for upregulation of spontaneously active histamine H2 receptors. Proc Natl Acad Sci U S A.

[68] Page DM, Wittamer V, Bertrand JY, Lewis KL, Pratt DN, Delgado N (2013). An evolutionarily conserved program of B-cell development and activation in zebrafish. Blood.

[69] David S, Zarruk JG, Ghasemlou N (2012). Inflammatory pathways in spinal cord injury. Int Rev Neurobiol.

[70] MacRae CA, Peterson RT (2022). Zebrafish as a Mainstream Model for *In vivo* Systems Pharmacology and Toxicology. Annu Rev Pharmacol Toxicol.

[71] Baraban SC, Dinday MT, Hortopan GA (2013). Drug screening in Scn1a zebrafish mutant identifies clemizole as a potential Dravet syndrome treatment. Nat Commun.

[72] Oprişoreanu A-M, Smith HL, Krix S, Chaytow H, Carragher N, Gillingwater TH (2021). Automated *in vivo* drug screening for synapse-stabilisation in zebrafish. Dis Model Mech.

[73] Chio JCT, Wang J, Surendran V, Li L, Zavvarian MM, Pieczonka K (2021). Delayed administration of high dose human immunoglobulin G enhances recovery after traumatic cervical spinal cord injury by modulation of neuroinflammation and protection of the blood spinal cord barrier. Neurobiol Dis.

[74] Shen H, Xu B, Yang C, Xue W, You Z, Wu X (2022). A DAMP-scavenging, IL-10-releasing hydrogel promotes neural regeneration and motor function recovery after spinal cord injury. Biomaterials.

[75] Coll-Miró M, Francos-Quijorna I, Santos-Nogueira E, Torres-Espin A, Bufler P, Dinarello CA (2016). Beneficial effects of IL-37 after spinal cord injury in mice. Proc Natl Acad Sci U S A.

[76] Miao W, Zhao Y, Huang Y, Chen D, Luo C, Su W (2020). IL-13 Ameliorates Neuroinflammation and Promotes Functional Recovery after Traumatic Brain Injury. J Immunol.

[77] White SV, Czisch CE, Han MH, Plant CD, Harvey AR, Plant GW (2016). Intravenous Transplantation of Mesenchymal Progenitors Distribute Solely to the Lungs and Improve Outcomes in Cervical Spinal Cord Injury. Stem Cells.

[78] Badner A, Hacker J, Hong J, Mikhail M, Vawda R, Fehlings MG (2018). Splenic involvement in umbilical cord matrix-derived mesenchymal stromal cell-mediated effects following traumatic spinal cord injury. J Neuroinflammation.

[79] Cheng Z, Bosco DB, Sun L, Chen X, Xu Y, Tai W (2017). Neural Stem Cell-Conditioned Medium Suppresses Inflammation and Promotes Spinal Cord Injury Recovery. Cell transplantation.

[80] Beresford J, Colin-Jones DG, Flind AC, Langman MJ, Lawson DH, Logan RF (1998). Postmarketing surveillance of the safety of cimetidine: 15-year mortality report. Pharmacoepidemiol Drug Saf.

[81] de Sousa N, Pinho AG, Monteiro S, Liberato V, Santos DJ, Campos J (2022). Acute Baclofen administration promotes functional recovery after spinal cord injury. Spine J.

[82] Yu CG, Bondada V, Iqbal H, Moore KL, Gensel JC, Bondada S (2021). Inhibition of Bruton Tyrosine Kinase Reduces Neuroimmune Cascade and Promotes Recovery after Spinal Cord Injury. Int J Mol Sci.

[83] Saghazadeh A, Rezaei N (2017). The role of timing in the treatment of spinal cord injury. Biomed Pharmacother.

[84] McCarthy DM (1983). Ranitidine or cimetidine. Ann Intern Med.

[85] Starkey ML, Schwab ME (2012). Anti-Nogo-A and training: can one plus one equal three?. Exp Neurol.

[86] Wang X, Zhou T, Maynard GD, Terse PS, Cafferty WB, Kocsis JD (2020). Nogo receptor decoy promotes recovery and corticospinal growth in non-human primate spinal cord injury. Brain.

[87] Lang BT, Cregg JM, DePaul MA, Tran AP, Xu K, Dyck SM (2015). Modulation of the proteoglycan receptor PTPσ promotes recovery after spinal cord injury. Nature.

[88] Francos-Quijorna I, Sánchez-Petidier M, Burnside ER, Badea SR, Torres-Espin A, Marshall L (2022). Chondroitin sulfate proteoglycans prevent immune cell phenotypic conversion and inflammation resolution via TLR4 in rodent models of spinal cord injury. Nat Commun.

[89] Hellal F, Hurtado A, Ruschel J, Flynn KC, Laskowski CJ, Umlauf M (2011). Microtubule stabilization reduces scarring and causes axon regeneration after spinal cord injury. Science.

