# Supplementary material for: Drug screening in zebrafish larvae reveals inflammation-related modulators of secondary damage after spinal cord injury in mice
Source: Theranostics. 2023 Apr 23;13(8):2531–51. doi: 10.7150/thno.81332 (PMC10196818; doi:10.7150/thno.81332)
Supplement: Supplementary file 1 — Supplementary figures and table. [file thnov13p2531s1.pdf]

Supplemental Information

Drug screening in zebrafish larvae reveals inflammation-related modulators of secondary damage after spinal cord injury in mice

Ana-Maria Oprişoreanu, Fari Ryan, Claire Richmond, Yuliya Dzekhtsiarova, Neil O. Carragher, Thomas Becker, Samuel David,Catherina G. Becker

11 Figures + 1 Table

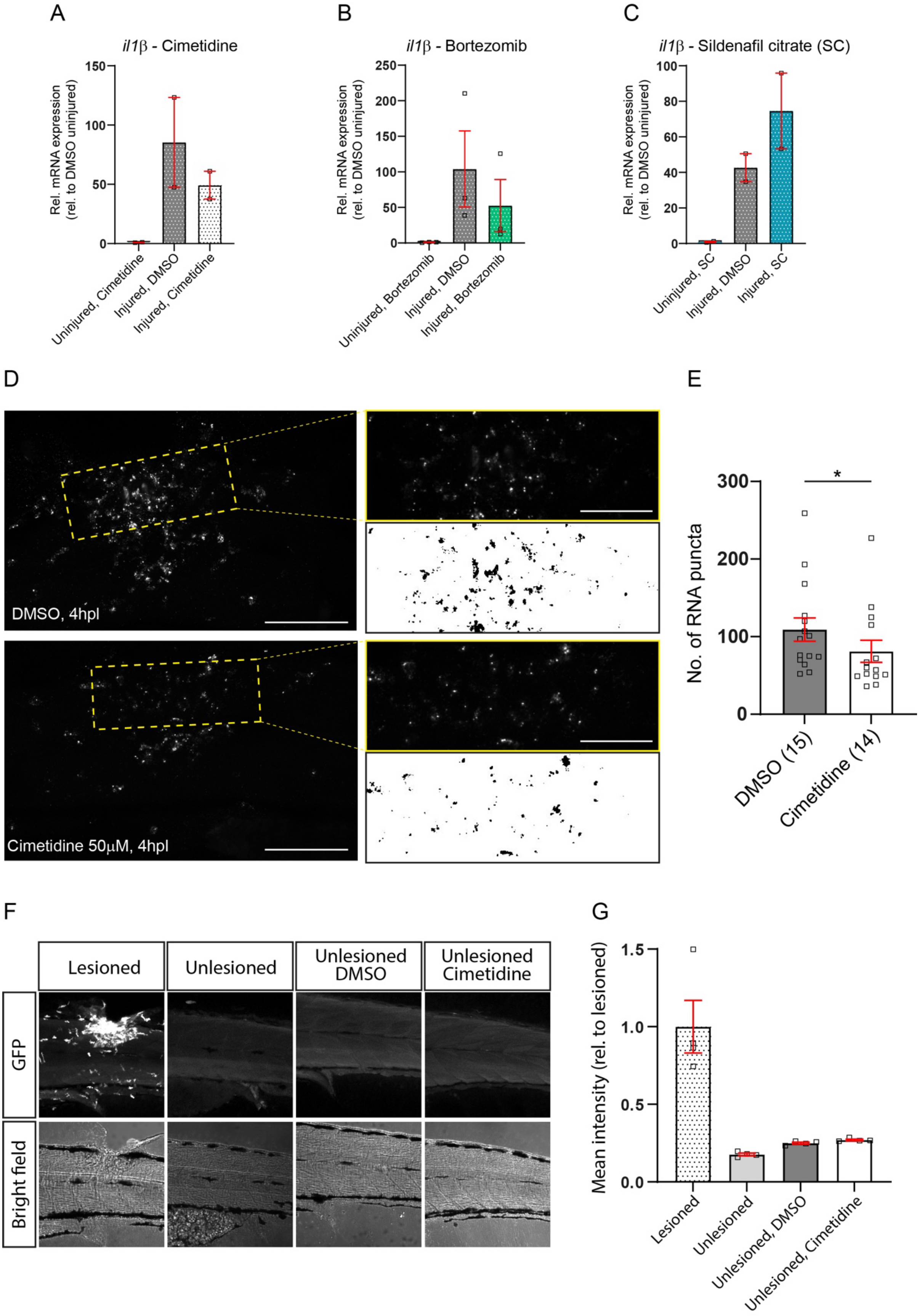
A
il1β - Cimetidine
Rel. mRNA expression (rel. to DMSO uninjured)
Uninjured, Cimetidine
Injured, DMSO
Injured, Cimetidine
B
il1β - Bortezomib
Uninjured, Bortezomib
Injured, DMSO
Injured, Bortezomib
C
il1β - Sildenafil citrate (SC)
Uninjured, SC
Injured, DMSO
Injured, SC
D
DMSO, 4hpl
Cimetidine 50μM, 4hpl
E
No. of RNA puncta
DMSO (15)
Cimetidine (14)
F
Lesioned
Unlesioned
Unlesioned DMSO
Unlesioned Cimetidine
GFP
Bright field
G
Mean intensity (rel. to lesioned)
Lesioned
Unlesioned
Unlesioned, DMSO
Unlesioned, Cimetidine

Figure S1: Abundance of *il-1β* mRNA is reduced by cimetidine and bortezomib treatment after spinal injury in larval zebrafish. **A,B:** Relative *il-1β* mRNA level is decreased in larvae treated with cimetidine and bortezomib. **C:** Sildenafil citrate did not reduce the *il-1β* mRNA level. For each data point, 50 wild-type zebrafish larvae were pooled. The level of mRNA was measured at 4 hpl. **D**: An *il-1β* HCR labeling on 3 dpf larvae is shown. Lateral views of the injury site in vehicle- and cimetidine-treated larvae at 4 hpl are shown. The yellow box depicts the region of interest at the injury site that is used in puncta quantification (H: 75 μm, W: 200 μm, Depth: 50 μm). Scale bar: 100 μm and 50 μm (yellow box). **E**: Quantification of the RNA puncta detected by HCR. The number of RNA puncta/clusters is decreased by cimetidine treatment (Mann-Whitney test, p* = 0.0423). **F:** Lateral view of injured and injured and uninjured *il-1β*:GFP larvae at 4 dpf are shown. **G:** Quantification of the average GFP fluorescence in lesioned and unlesioned larvae at 4 dpf. Unlesioned larvae show no specific GFP fluorescence compared to lesioned control.

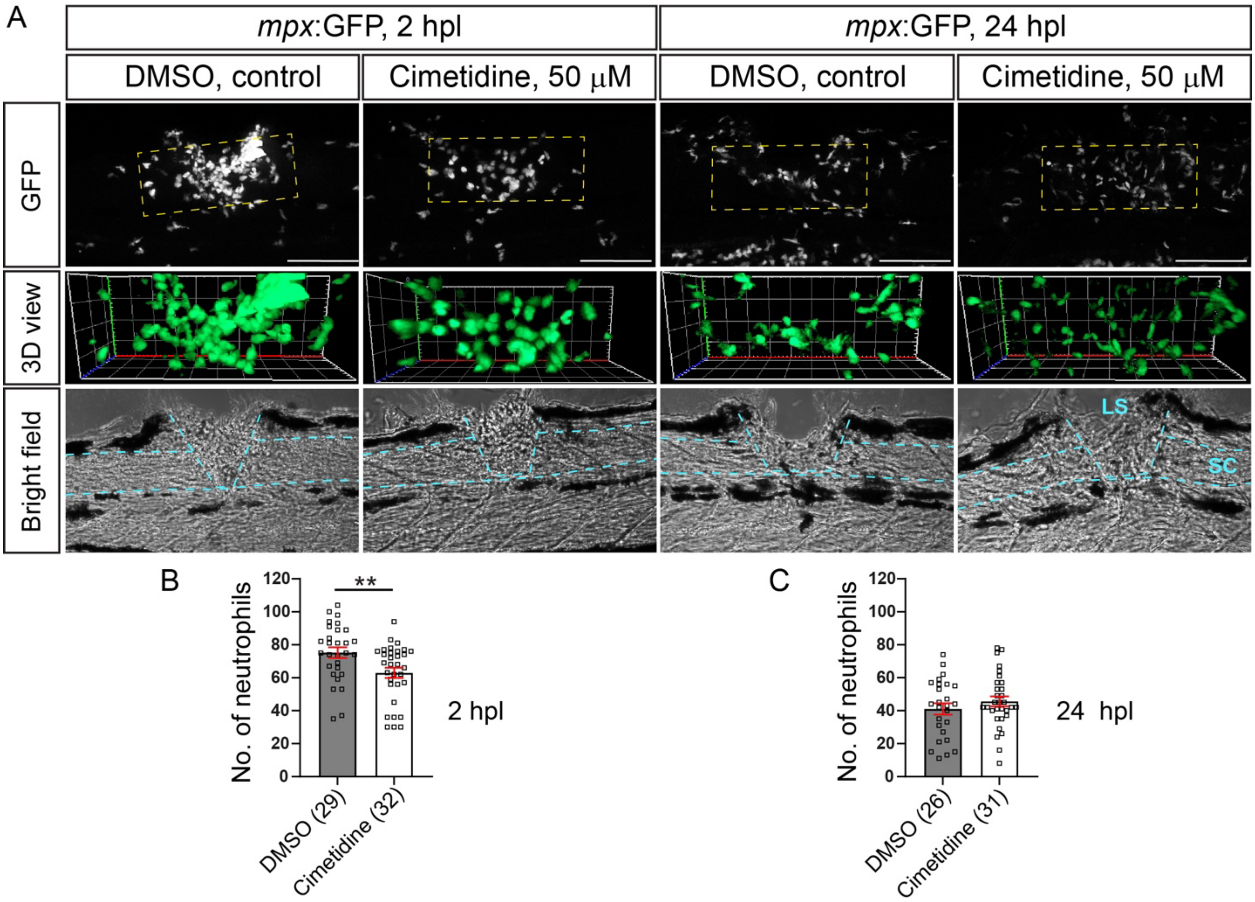

Figure S2: Cimetidine reduces the number of *mpx*:GFP+ neutrophils in the injury site. **A:** Representative images of the lateral view of the injury site in the *mpx*:GFP line treated with cimetidine and DMSO-control are shown. **B:** The number of neutrophils is reduced at 2 hpl in the Cimetidine group (t-test, p** = 0.0076). **C:** The number of neutrophils is not altered by cimetidine treatment at 24 hpl.

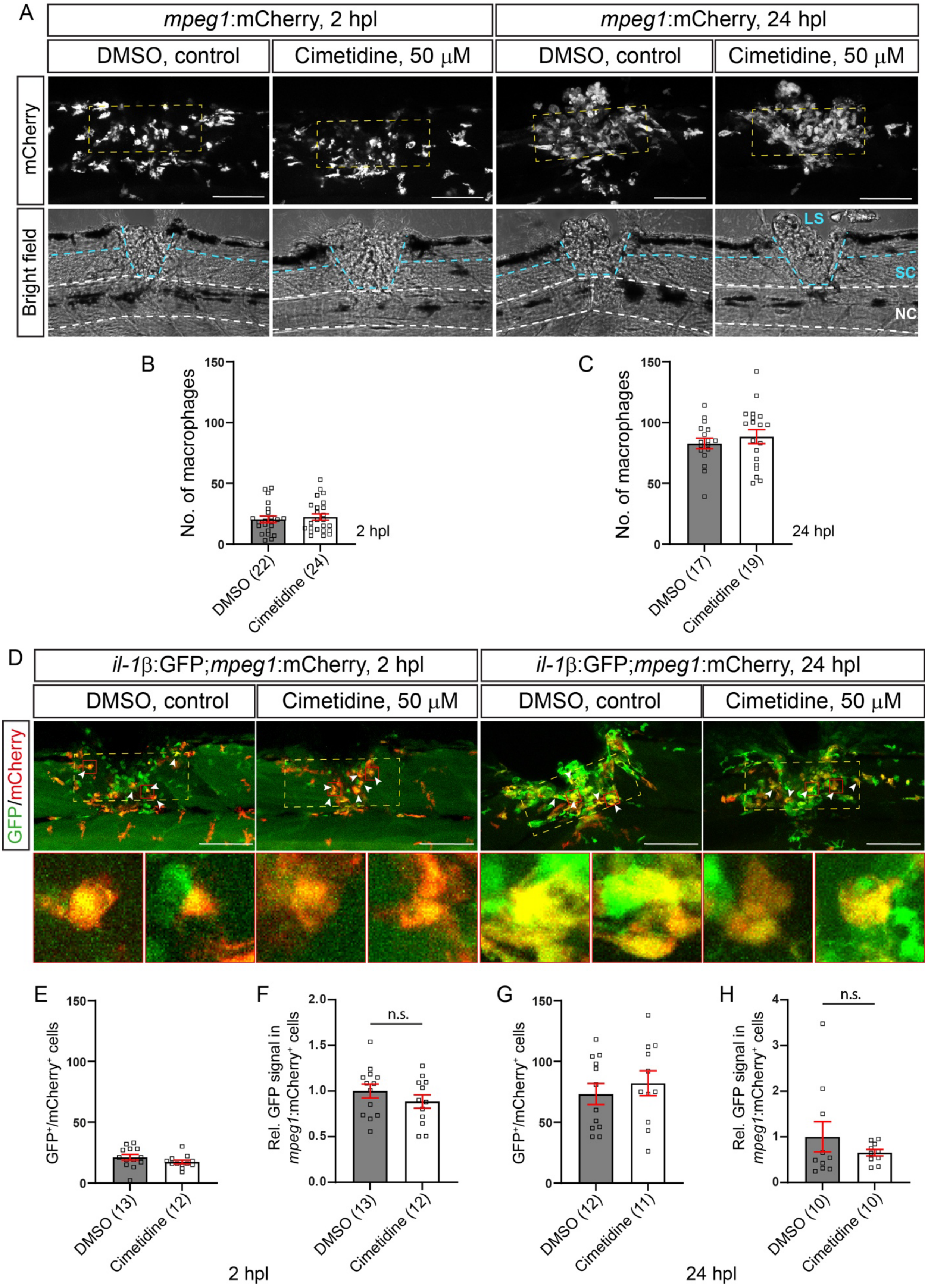

Figure S3: Cimetidine reduces *il-1β*:GFP reporter gene activity in macrophages/microglia. **A**: Representative lateral views of the injury site in which

macrophages are labelled in the *mpeg1*:mCherry line are shown. The yellow box depicts the area of quantification. In the bright-field images, the notochord (NC), spinal cord (SC) and lesion site (SC) are depicted. Scale bar: 100 µm. **B,C**: The overall number of macrophages is not altered at 2 or 24 hpl by Cimetidine treatment. **D**: Lateral views of the injury site in *il-1β*:GFP; *mpeg1*:mCherry double-transgenic larvae are shown. Yellow box depicts the area of quantification. Lower row shows higher magnification images of macrophages. Arrowheads indicate some individual cells. Scale bar: 100 µm. **E**: The number of double-positive macrophages (GFP^+/$mCherry^+$) cells is not altered by cimetidine treatment at 2hpl. **F**: At 2 hpl the relative GFP fluorescence in *mpeg1*:$mCherry^+$ cells is unchanged following Cimetidine treatment. **G**: At 24 hpl, the number of double-positive macrophages (GFP^+/$mCherry^+$) is not altered by Cimetidine treatment. **H**: The relative GFP signal shows a non-significant trend to reduced expression of the *il-β* transgene fluorescence in *mpeg1*:GFP^+ cells at 24 hpl (one sample t-test, p*** = 0.0008).

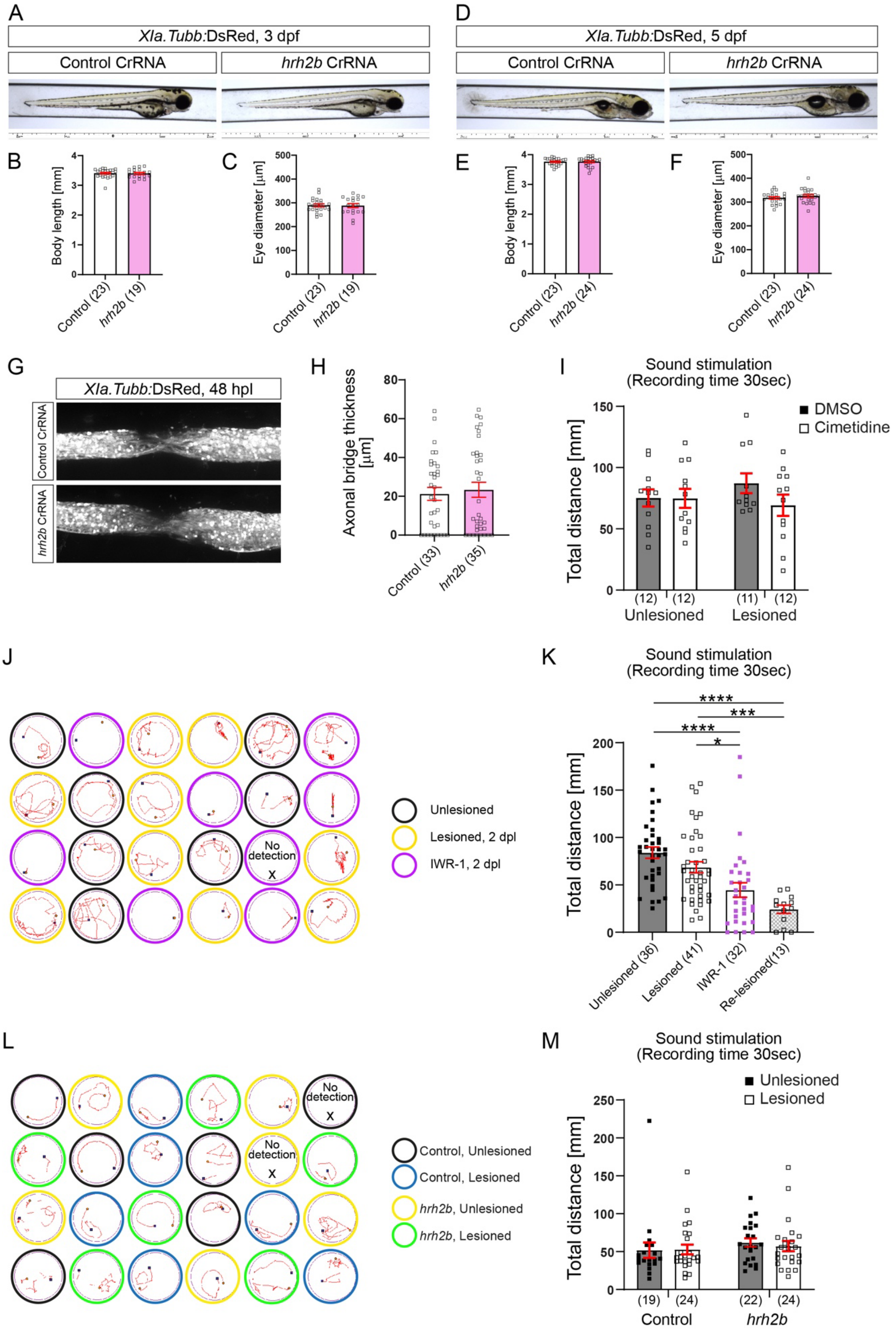

Figure S4: Somatic mutation of *hrh2b* crispants does not impair overall development or axonal regeneration. **A**: Images of 3 dpf control and *hrh2b* haCR-injected larvae.

B,C: Body length and eye diameter is not altered in *hrh2b* haCR-injected larvae. **D**: Images of 5 dpf control and *hrh2b* haCR-injected larvae are shown. **E,F**: Body length and eye diameter is not altered in *hrh2b* haCR-injected larvae. **G**: Representative images of bridged spinal cord following injury in control and *hrh2b* haCR-injected larvae. **H**: Quantification of the axonal bridge thickness shows no difference between control and injected *hrh2b* haCRs. **I**: Cimetidine treatment does not alter the total swim distance in unlesioned and lesioned control animals (two-way ANOVA, interaction F(1, 43) = 1,232, p 0 0,2732) **J,K**: Total swim distance after spinal lesion shows no difference between uninjured and recovered larvae 2 days after spinal injury at 3 dpl, but is reduced by IWR-1 control treatment in lesioned larvae, indicating sensitivity of the measurements. Total swim distance (vibration stimulation) is decreased in lesioned IWR-1 and re-lesioned control groups in comparison to unlesioned group (Kruskal-Wallis test p****< 0.0001 with Dunn's multiple comparison test: unlesioned vs IWR-1 p****< 0.0001; unlesioned vs re-lesioned p****< 0.0001; lesioned vs IWR-1 p* = 0.0169; lesioned vs re-lesioned p*** = 0.0008). **L,M**. Total swim distance between control and *hrh2b* haCR-injected larvae shows no statistically significant differences (two-way ANOVA, interaction F(1, 85) = 0,1538, p = 0,6960).

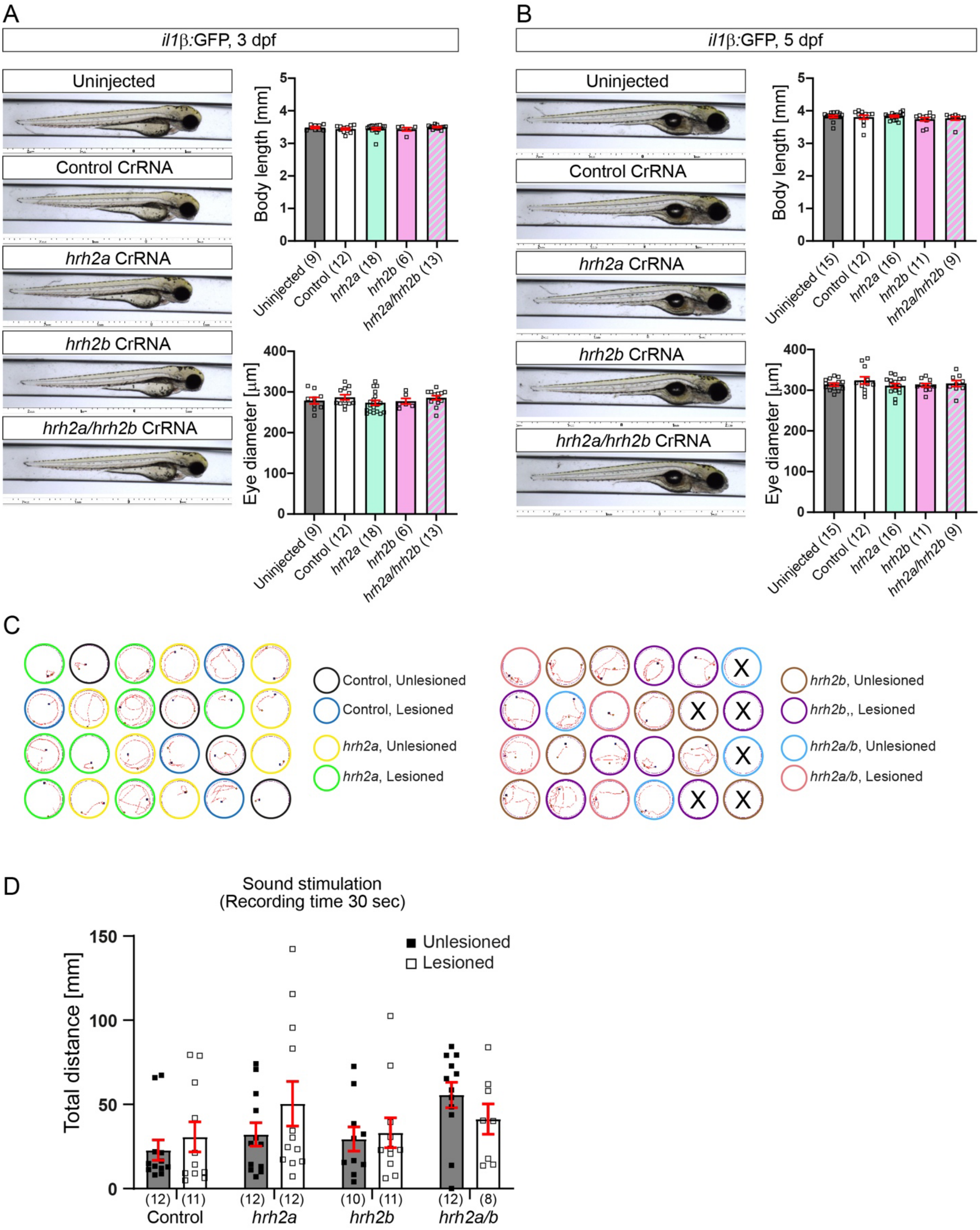

Figure S5: Combined *hrh2a/b* somatic mutations do not affect overall development and recovery of swimming capacity. **A,B:** Representative images of 3 dpf and 5 dpf larvae injected with different *hrh2* haCRs are shown. Body length and eye diameter is not altered in single or double haCR-injected larvae in comparison to uninjected and control CrRNA-injected larvae at both time points. **C**: Examples of the complete

swim path in *hrh2* haCR-injected larvae are shown. Different conditions are depicted with different colored circles. **D**. Quantification of the total swim distance shows no significant difference between groups [two-way ANOVA, $p = 0.3434$, $F(3, 80) = 1.127$]. Swimming behavior was measured at 5 dpf.

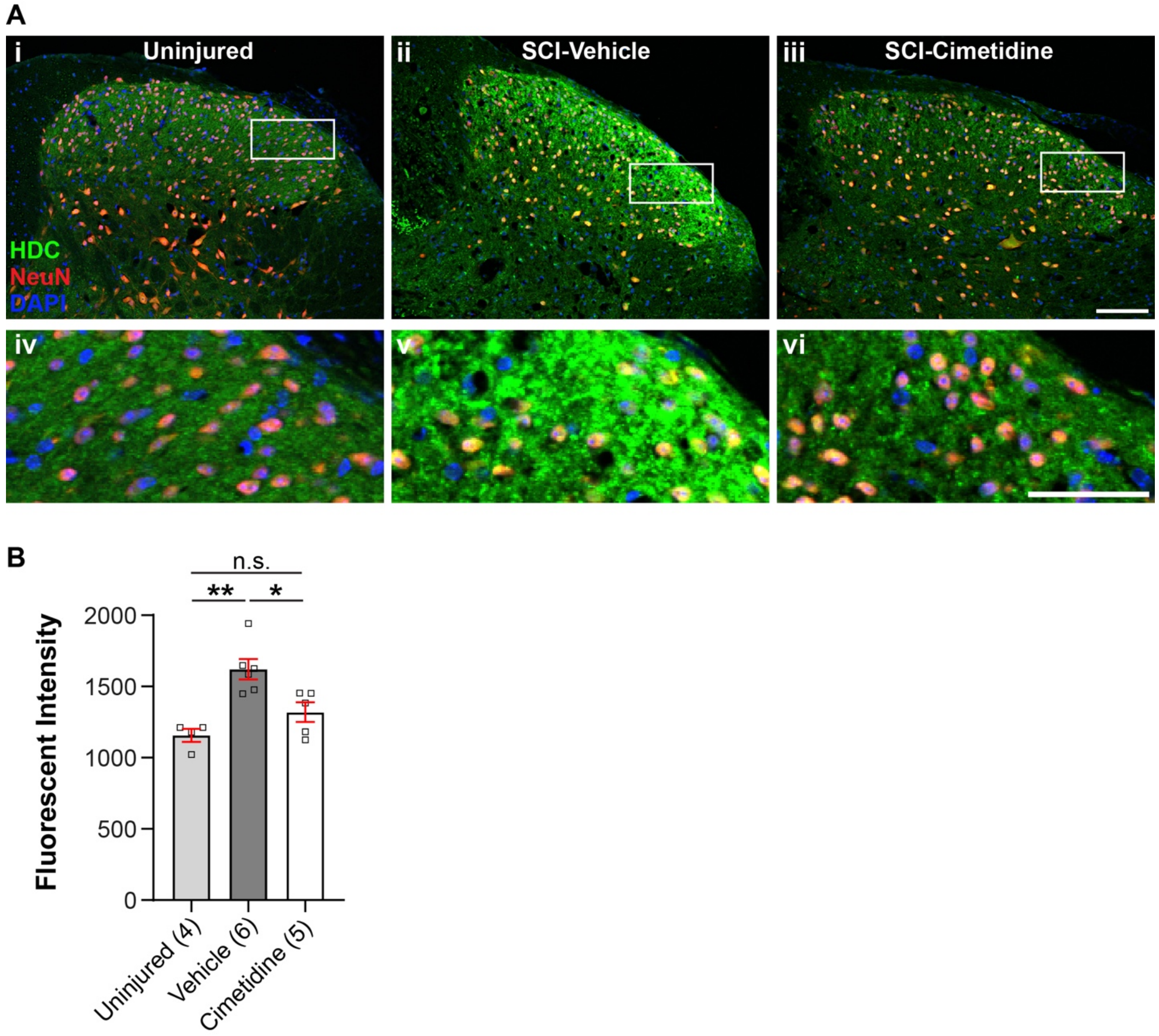

Figure S6: Cimetidine reduces HDC expression in the injured mouse spinal cord. **A:** Double immunofluorescence labeling of HDC and NeuN shows HDC labeling is barely detectable in the uninjured spinal cord (Ai, iv) and is markedly increased 48h after SCI (ii, v). In contrast, Cimetidine treatment for 48 h after SCI reduces this staining substantially (Aiii, vi). Scale bar in Aiii = 100µm; and Avi = 50 µm. **B**: Quantification by densitometry shows significant increase in HDC labeling 48h after SCI compared to uninjured values, and the significant reduction by Cimetidine treatment compared to SCI-vehicle controls. One-way ANOVA with post-hoc Tukey

multiple comparison test: uninjured vs. vehicle: $p^{**} = 0.0013$; uninjured vs. cimetidine: $p = 0.2829$; vehicle vs. cimetidine: $p = 0.01666$; n = 4 - 6 mice per group.

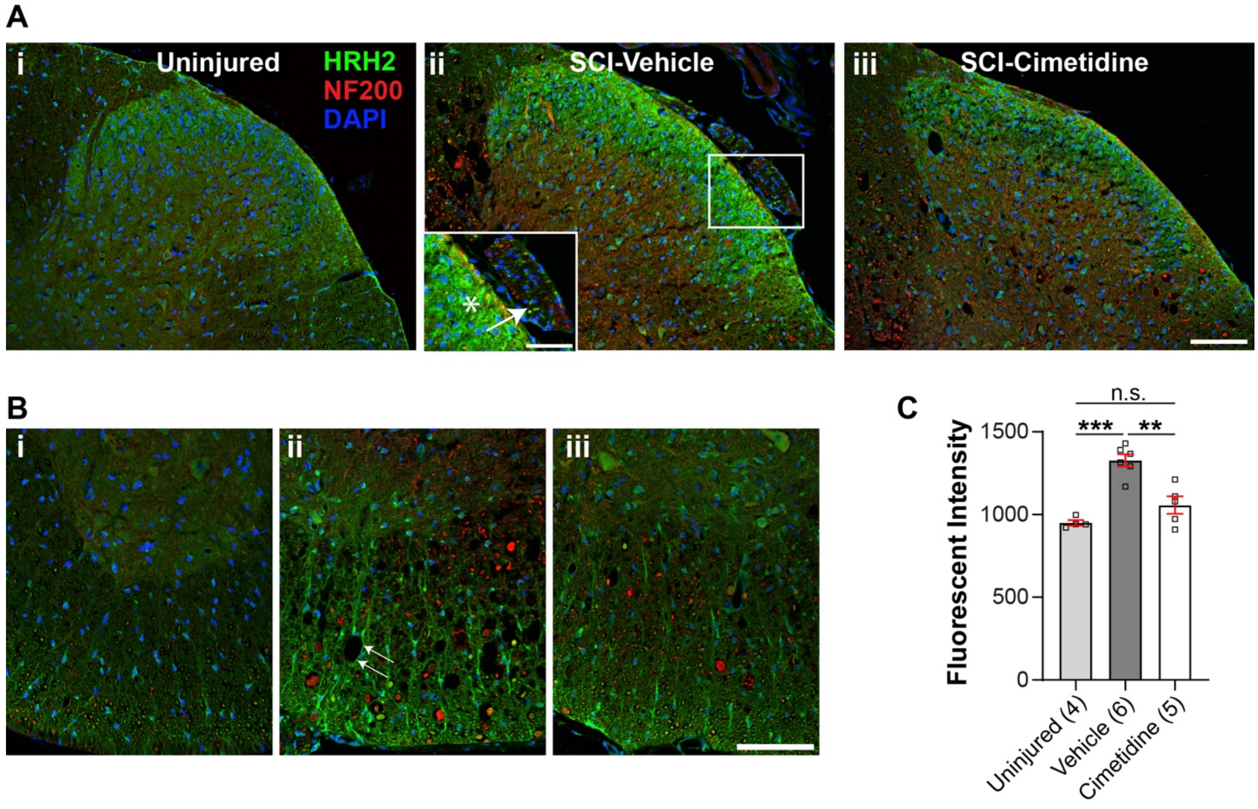

Figure S7: Cimetidine reduces HRH2 expression in the injured mouse spinal cord. **A:** Immunofluorescence labeling shows HRH2 is expressed at very low levels in the dorsal horn in the uninjured spinal cord (Ai). Immunofluorescence is markedly increased 48h after SCI (ii); Inset shows higher magnification of the area shown in white rectangle in panel ii. Note labeling of some NF200-negative axons (red) in the dorsal root, which could be small unmyelinated fibers that lack NF (arrow). Note the strong HRH2 labeling in the superficial later of the dorsal horn (asterisk inset). In contrast, Cimetidine treatment for 48h after SCI markedly reduces this staining (Aiii). **B.** As compared to the minimal immunolabeling of HRH2 in uninjured mouse spinal cord (Bi), there is a marked increase in HRH2 immunoreactivity along blood vessels (arrows) in the ventral and lateral regions of the spinal cord 48h after SCI (Bii). This increase is reduced by Cimetidine treatment (Biii). Scale bars in Aiii and Biii = 100µm; inset = 50µm. **C.** Quantification by densitometry shows significant increase in HRH2 labeling 48h after SCI compared to uninjured values, and the significant reduction by Cimetidine treatment compared to SCI-vehicle controls. One-way ANOVA with post-hoc Tukey multiple comparison test: uninjured vs. vehicle: p^{***} =

0.0001; uninjured vs. cimetidine: $p = 0.2349$; vehicle vs. cimetidine: $p^{**} = 0.0011$; n = 4-6 mice per group.

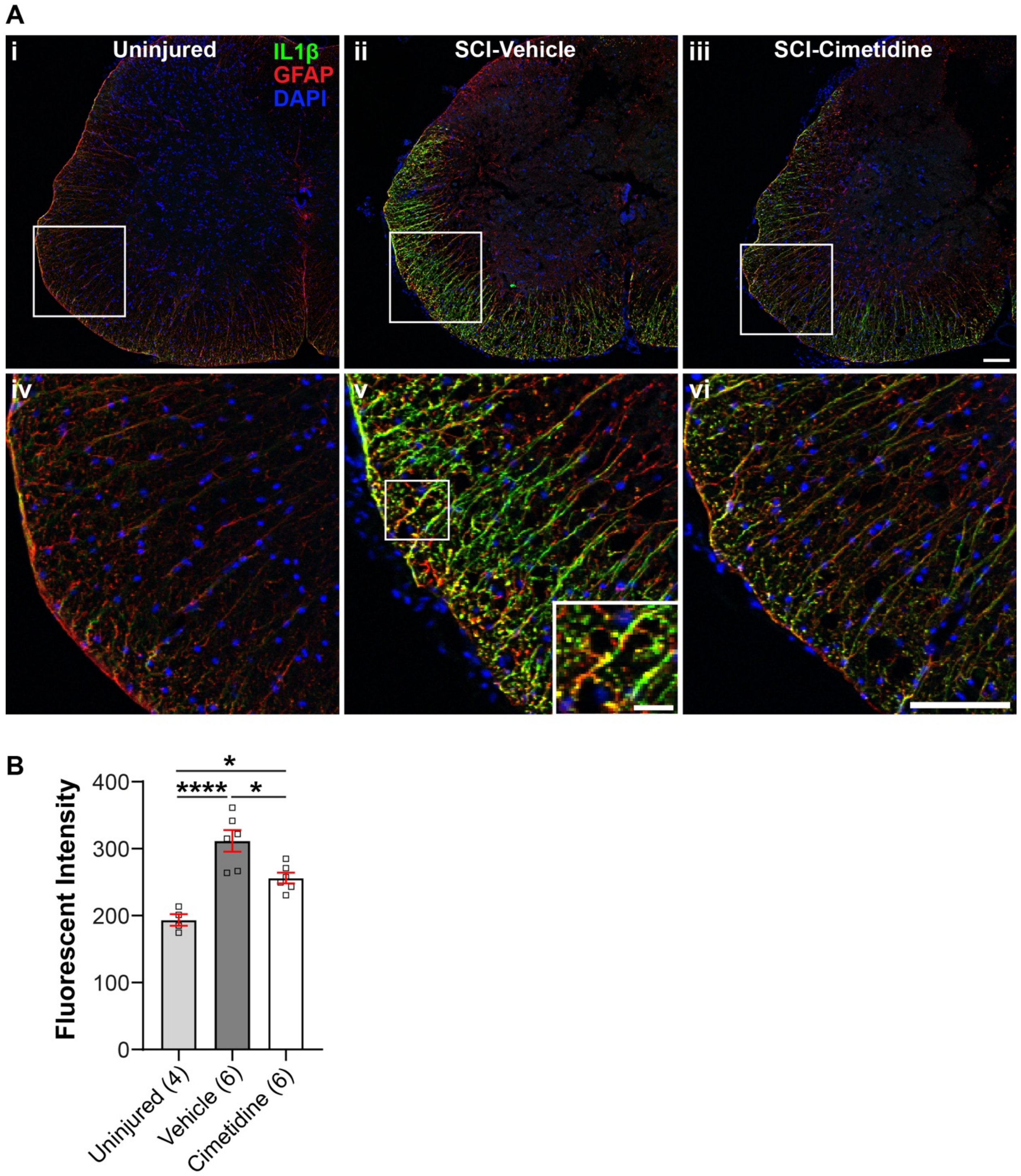

Figure S8: Cimetidine reduces IL-1β expression in the injured mouse spinal cord. **A:** Double immunofluorescence labeling with anti-IL-1β (green) and anti-GFAP (red) antibodies of uninjured (Ai, iv), 48h after SCI treated with vehicle (Aii, v) and 48h after SCI treated with Cimetidine (Aiii, vi) are shown. The area in the white rectangles shown in the low magnification images (Ai-iii) are shown in higher magnification in Aiv-vi. Note the increased expression of IL-1β in GFAP+ astrocytes

48 h after SCI (Aii, v), and the reduction of this after Cimetidine treatment (Aiii, vi). Inset in panel v shows higher magnification of the area in rectangle. Note the double labeling of IL-1β and GFAP. Scale bars in Aiii and vi = 100μm; inset = 20μm. **B:** Quantification by densitometry shows significant increase in IL-1β labeling 48h after SCI compared to uninjured values, and the significant reduction by Cimetidine treatment compared to SCI-vehicle controls. This reduction does not reach uninjured levels. One-way ANOVA with post-hoc Tukey multiple comparison test: uninjured vs. vehicle: $p^{****} < 0.0001$; uninjured vs. cimetidine: $p^{*} = 0.0126$; vehicle vs. cimetidine: $p^{*} = 0.0130$; n = 4-6 mice per group.

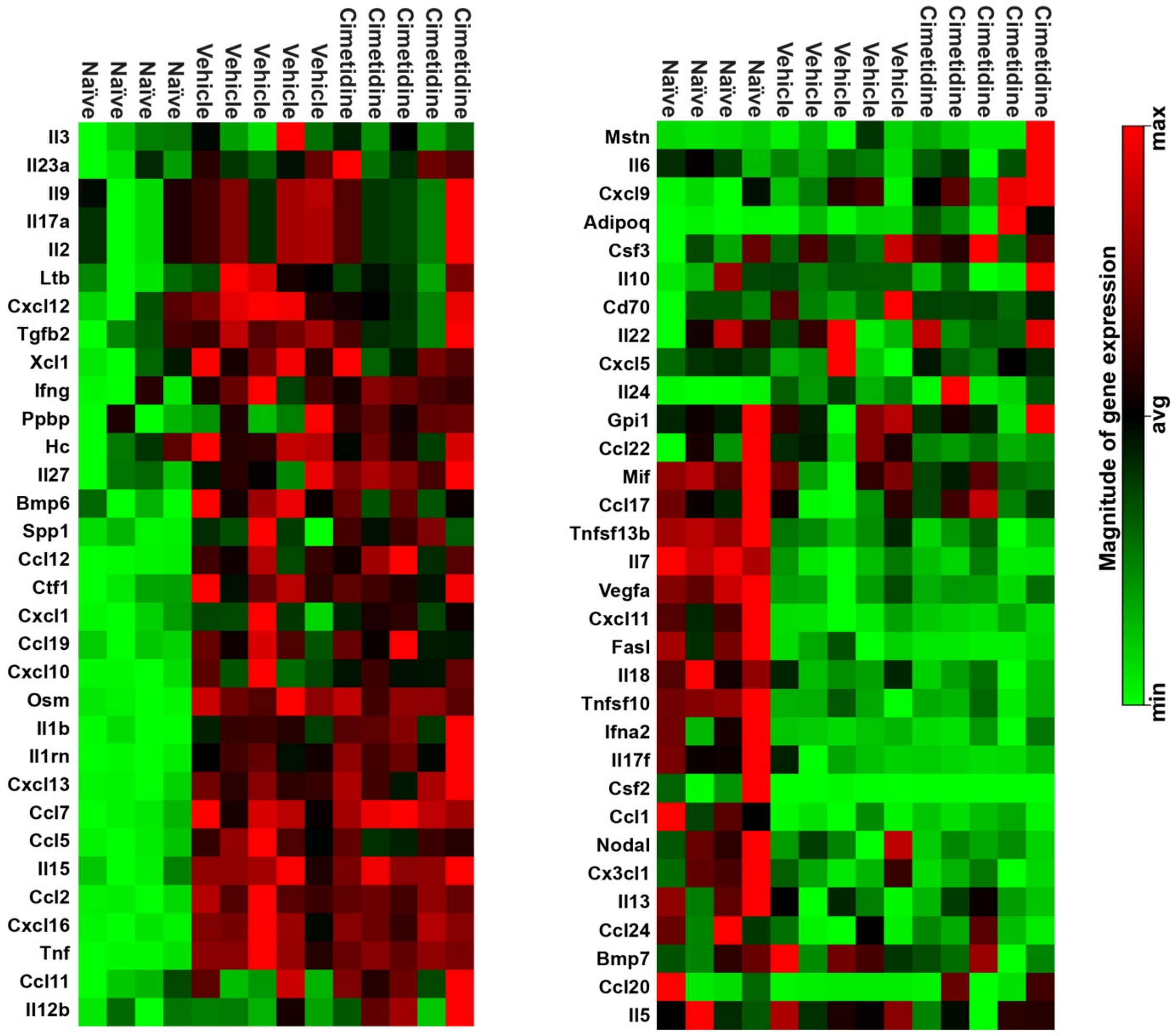

Figure S9: Heat map of results of the 64 genes that did not show statistically significant differences between Cimetidine and Vehicle treated groups. RT^2 profiler cytokine array (PAMM-150Z) after 10 days of Cimetidine treatment; decreased (green) and increased (red) transcript levels in individual mice in the three indicated experimental groups is shown.

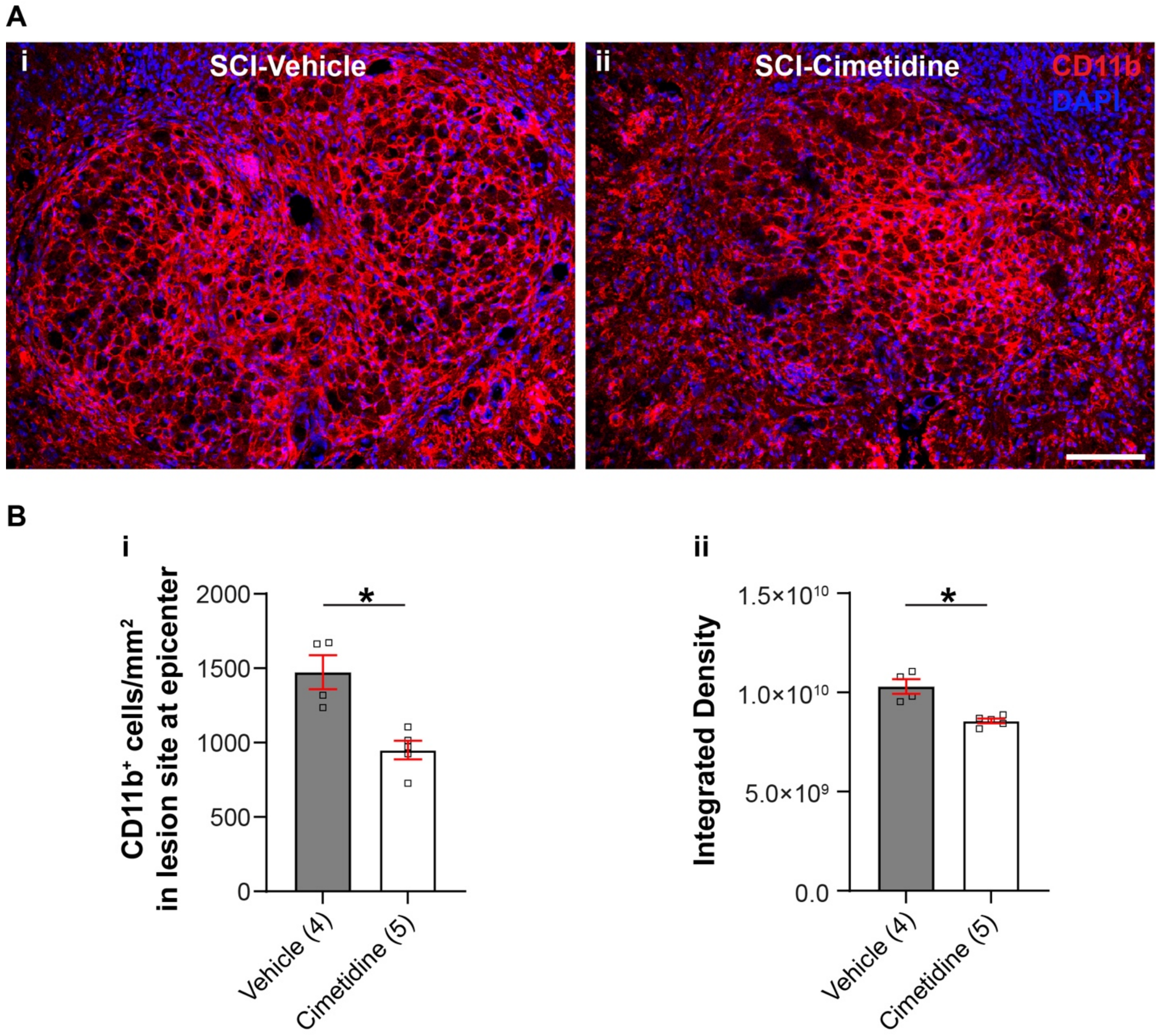

Figure S10: Cimetidine reduces CD11b+ macrophages/microglia in the injured mouse spinal cord. A: CD11b immunostaining at the lesion center 4 weeks after SCI in vehicle treated (i) and Cimetidine treated (ii) mice is shown. Note the marked reduction in CD11b staining in the Cimetidine treated animal. Scale bar = 100µm. **B:** Quantification of the number of CD11b+ cell and integrated CD11b fluorescence density, both show significant reductions in the Cimetidine treated group. vehicle vs. cimetidine: two-tailed Mann Whitney U-test, cell number: p^* = 0.0159; integrated density: p^* = 0.0159; n = 4-5 mice per group.

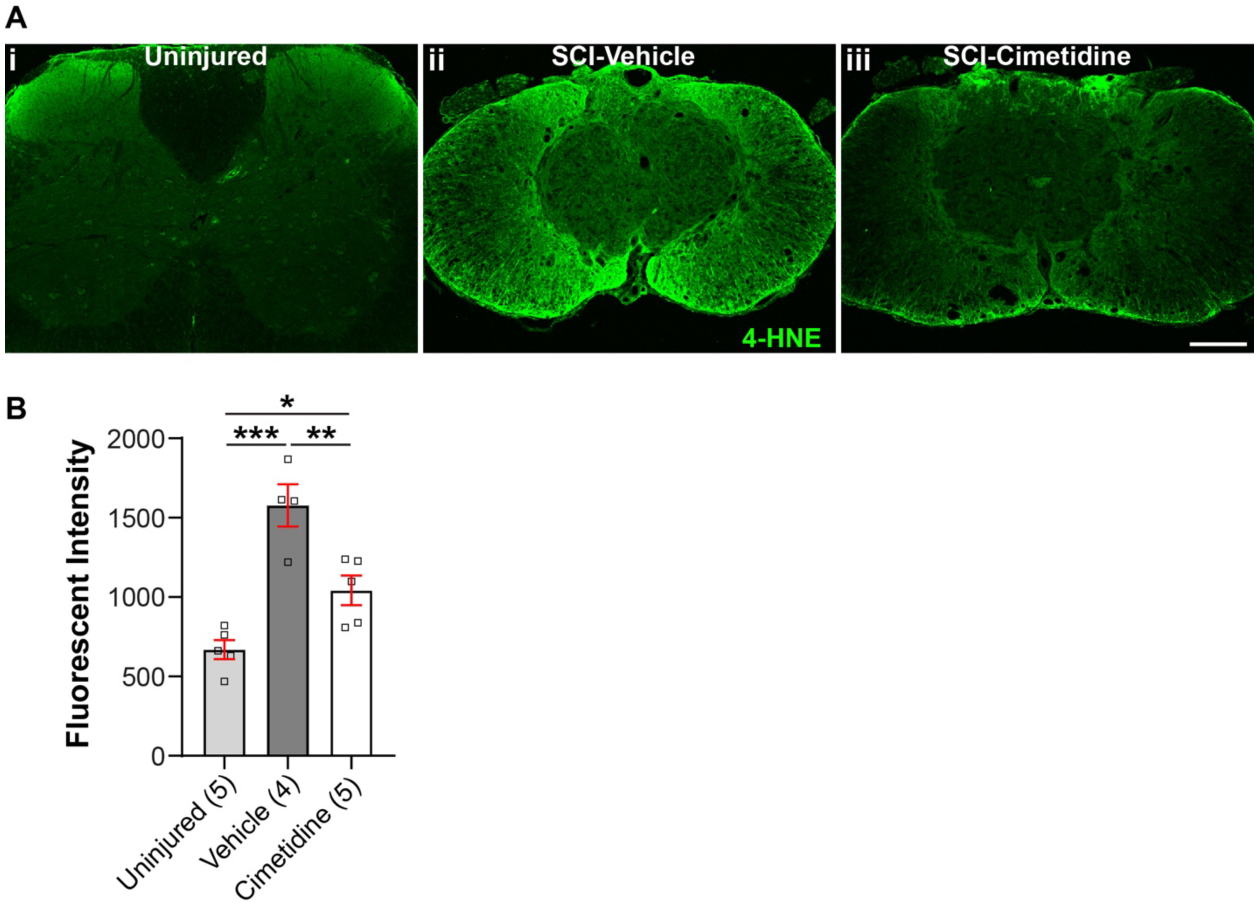

Figure S11: Cimetidine reduces 4HNE labeling in the injured mouse spinal cord. **A:** Immunofluorescence labeling with anti-4HNE antibody shows barely detectable staining in normal, uninjured mice (i). Labeling is markedly increased at the lesion epicenter 4 weeks after SCI (ii) and reduces substantially after Cimetidine treatment (iii). Scale bar in Aiii = 200 μm. **B:** Quantification of the fluorescence in the three groups is shown. Note the statistically significant increase in vehicle-treated SCI group compared to uninjured controls, and a significant reduction in the Cimetidine-treated SCI group. One-way ANOVA with post-hoc Tukey multiple comparison test: uninjured vs. vehicle: $p^{***} = 0.0001$; uninjured vs. cimetidine: $p^{*} = 0.0362$; vehicle vs. cimetidine: $p^{**} = 0.0064$; n = 4 - 5 mice per group.

Table S1: List of tested compounds during various screening steps. From a total of 1081 tested compounds, 101 entered the screening step. After applying the 0.5 threshold limit (50% reduction in overall GFP fluorescence) only 33 compounds entered the validation step, from which only 5 entered the mechanistic step (qRT-PCR). Out of this 5, only 3 compounds induced a decrease in the *il-1β* mRNA level.

Compound Name	101 compounds in Screening step	33 compounds in Validation step	5 compounds in Mechanistic step
((-)-epicatechin gallate	0.418906	0.151689546	no effect
Bortezomib	0.33639	0.132166303	Approx. 70% decrease (10uM)
Cimetidine	0.486441	0.468904757	Approx. 40% decrease (10uM)
Ozanimod	0.143326	0.498244702	5x increase
Sildenafil Citrate	0.381337	0.498517192	40% decrease (10uM)
Doxepin (hydrochloride)	0.514449		
Haloanalyzedsol Propionate	0.521749		
Trospium Chloride	0.330373	0.593479874	
Meropenem	0.45261	0.59897679	
Anagrelide HCl	0.371969	0.612476393	
Cilostazol	0.400504	0.686921936	
Thioguanine (6-Thioguanine)	0.206254	0.712436374	
Desloratadine	0.3183	0.7200	
CORM-3	0.215168	0.76429397	
Fluvastatin·Na	0.5652		
Chlorpheniramine Maleate	0.31188	0.821362921	
Adapalene	0.479656	0.828239678	
Raltegravir	0.213463	0.830261145	
Streptozocin	0.405402	0.879140584	
Zileuton	0.205783	0.911006413	
Ganciclovir	0.4151	0.9300	
Sodium salicylate	0.420673	0.97841957	
Gallic acid	0.513168		
Eplerenone	0.457236	1.019261985	
Griseofulvin	0.4733	1.0281	
Cladribine	0.471537	1.063525319	
Isocarboxazid	0.4843	1.0755	
Cidofovir	0.500498	1.08680294950974	
Triamcinolone	0.517687		
MCC950 sodium	0.594586		
Levocetirizine Dihydrochloride	0.3005	1.17912852	
Lacosamide	0.3708	1.1794	

Homatropine Methylbromide	0.2566	1.186600197	
Doxylamine Succinate	0.511141		
Masitinib	0.38486	1.22040028	
Norepinephrine Bitartrate Monohydrate	0.523672		
Apremilast	0.436108	1.244042901	
Mianserin HCl	0.396956	1.290897615	
Diphenylpyraline HCl	0.427619	1.518950931	
Fosphenytoin·Na Pentahydrate	0.4504	1.6842	
Theophylline	1.118231		
Cyclophosphamide	0.4746	1.02814137101296	
Alprostadil	0.513304		
Estrone	0.5155		
SRT3190	0.539743		
Plerixafor	0.554341		
Avanafil	0.573995		
Amikacin Disulfate	0.577308		
Pemetrexed Disodium	0.581522		
Dyphylline	0.615409		
Celecoxib	0.615582		
Tetrahydrozoline HCl	0.624066		
Methylprednisolone	0.627563		
Epirubicin·HCl	0.634077		
Etidronate Disodium	0.6374		
Roflumilast	0.649022		
Cytarabine	0.6501		
Sulfasalazine	0.652941		
IRAK-1-4 Inhibitor I	0.653869		
Fluorometholone	0.6552		
Aminophylline	0.675427		
Temozolomide	0.682361		
Phenacetin	0.692716		
Acrivastine	0.694529		
Acemetacin	0.701233		
Mycophenolate Mofetil	0.714217		
Difluprednate	0.714532		
Dexrazoxane	0.720795		
Latanoprost	0.725187		
Cetirizine DiHCl	0.725441		
Tacrolimus	0.732574		
Darunavir	0.74007		
Bufexamac	0.747275		
Chlorpropamide	0.772882		

Dihydromyricetin	0.773306		
Busulfan	0.790154		
Lamivudine	0.793		
Tamsulosin·HCl	0.796077		
Flurandrenolide	0.8176		
Piroxicam	0.832685		
Tempol	0.840882		
Docetaxel (Taxotere)	0.84913		
Cefepime·HCl Hydrate	0.850132		
Erythromycin	0.900522		
Gabapentin	0.932078		
Enalapril	0.9692		
Tobramycin	0.969207		
Methotrexate	0.990157		
Primaquine Phosphate	1.008743		
Atovaquone	1.012252		
Megestrol Acetate	1.02385		
Fexofenadine·HCl	1.035		
Dexchlorpheniramine Maleate	1.130611		
Vecuronium Bromide	1.131693		
Antazoline HCl	1.142164		
Sasapyrine	1.252478		
Milrinone	1.253701		
Esomeprazole Potassium	1.352		
Azathioprine	1.640131		
Rifabutin	1.86199		
Dutasteride	1.993384		
